# Intrahepatic Transcriptional Signature Associated with Response to Interferon-α Treatment in the Woodchuck Model of Chronic Hepatitis B

**DOI:** 10.1371/journal.ppat.1005103

**Published:** 2015-09-09

**Authors:** Simon P. Fletcher, Daniel J. Chin, Lore Gruenbaum, Hans Bitter, Erik Rasmussen, Palanikumar Ravindran, David C. Swinney, Fabian Birzele, Roland Schmucki, Stefan H. Lorenz, Erhard Kopetzki, Jade Carter, Miriam Triyatni, Linta M. Thampi, Junming Yang, Dalal AlDeghaither, Marta G. Murredu, Paul Cote, Stephan Menne

**Affiliations:** 1 Pharma Research & Early Development, Hoffmann-La Roche, Inc., Nutley, New Jersey, United States of America; 2 Roche Pharma Research & Early Development, Roche Innovation Center Penzberg, Penzberg, Germany; 3 Roche Pharma Research & Early Development, Roche Innovation Center Basel, Basel, Switzerland; 4 Department of Microbiology & Immunology, Georgetown University Medical Center, Washington, District of Columbia, United States of America; Albany Medical College, UNITED STATES

## Abstract

Recombinant interferon-alpha (IFN-α) is an approved therapy for chronic hepatitis B (CHB), but the molecular basis of treatment response remains to be determined. The woodchuck model of chronic hepatitis B virus (HBV) infection displays many characteristics of human disease and has been extensively used to evaluate antiviral therapeutics. In this study, woodchucks with chronic woodchuck hepatitis virus (WHV) infection were treated with recombinant woodchuck IFN-α (wIFN-α) or placebo (n = 12/group) for 15 weeks. Treatment with wIFN-α strongly reduced viral markers in the serum and liver in a subset of animals, with viral rebound typically being observed following cessation of treatment. To define the intrahepatic cellular and molecular characteristics of the antiviral response to wIFN-α, we characterized the transcriptional profiles of liver biopsies taken from animals (n = 8–12/group) at various times during the study. Unexpectedly, this revealed that the antiviral response to treatment did not correlate with intrahepatic induction of the majority of IFN-stimulated genes (ISGs) by wIFN-α. Instead, treatment response was associated with the induction of an NK/T cell signature in the liver, as well as an intrahepatic IFN-γ transcriptional response and elevation of liver injury biomarkers. Collectively, these data suggest that NK/T cell cytolytic and non-cytolytic mechanisms mediate the antiviral response to wIFN-α treatment. In summary, by studying recombinant IFN-α in a fully immunocompetent animal model of CHB, we determined that the immunomodulatory effects, but not the direct antiviral activity, of this pleiotropic cytokine are most closely correlated with treatment response. This has important implications for the rational design of new therapeutics for the treatment of CHB.

## Author Summary

Approximately 250 million people are chronically infected with HBV, and over 500,000 people die every year because of associated liver diseases. IFN-α has been used to treat patients with chronic HBV infection for over 20 years, but it is not well understood why some patients respond to treatment and others do not. In large part, this is because it is not practicable to obtain liver samples to characterize the intrahepatic response to IFN-α in patients with different treatment outcomes. In this study we used the woodchuck model of chronic HBV infection to study how IFN-α changes gene expression patterns in the liver during treatment. Surprisingly, we found that the treatment response did not correlate with the expression of antiviral effector genes that have previously been shown to mediate the direct antiviral effects of IFN-α in vitro. Instead, we found that the response to IFN-α treatment was associated with the presence of select immune cells (natural killer cells and T cells) in the liver. Our work also indicates that these immune cells inhibit the virus by killing infected cells, as well as in ways that do not require killing of liver cells. Altogether, our study suggests that new therapies that stimulate these immune cells in the liver may hold promise for the treatment of chronic HBV infection.

## Introduction

Approximately 250 million individuals live with chronic hepatitis B (CHB), and over half a million people are estimated to die each year due to CHB-associated liver diseases, such as cirrhosis and hepatocellular carcinoma (HCC) [1]. End-points of therapies for CHB are virological response (durable reduction in serum HBV DNA levels to a degree which varies by therapy), serological response (HBV e antigen (HBeAg) loss and seroconversion to anti-HBe in HBeAg-positive patients) and biochemical response (normalization of ALT levels). However, sustained loss of HBV surface antigen (HBsAg) off therapy is currently considered the ideal end-point. Recombinant interferon-α (IFN-α) is licensed for the treatment of CHB, but in contrast to potent nucleos(t)ides, virologic response is limited to a subset of patients [2]. Conversely, the rate of durable HBsAg loss is higher with IFN-α than with nucleos(t)ides, although still only occurs in <10% patients [2]. Despite more than two decades of clinical use, the mechanisms by which IFN-α controls HBV in responders are not well understood [3]. Defining the molecular basis for response remains an important goal, since mechanistic understanding of IFN-α activity could drive rational design of novel immunotherapeutic strategies and may lead to the identification of novel biomarkers of treatment response and/or patient stratification.

IFN-α is a pleiotropic cytokine that has both direct antiviral and immunomodulatory properties [4,5]. With regard to the former, IFN-α induces the expression of hundreds of interferon-stimulated genes (ISGs), many of which have antiviral effector functions [4]. Although the identification of key restriction factors has been challenging, various studies have indicated that IFN-α induces antiviral effectors of HBV. Most notably, the direct antiviral response to IFN-α has been demonstrated to inhibit the formation or accelerate the decay of replication-competent HBV capsids [6–9], inhibit virion secretion [10], reduce transcription from the viral genome (cccDNA; covalently closed circular DNA) [11,12], and to induce non-cytolytic degradation of cccDNA [13]. The direct antiviral activity of IFN-α is consistent with the reduction in viral antigen levels by high dose pegylated IFN-α in HBV-infected humanized mice that lack immune cells [14]. The immunomodulatory properties of IFN-α include activation of NK cells and B cells, as well as both direct and indirect activation of CD8^+^ T cell function [5,15]. Despite this potential to activate both innate and adaptive immunity, recent studies have revealed that IFN-α treatment boosts the number and function of NK cells in the periphery, but does not improve peripheral HBV-specific CD8^+^ T cells responses [16–19].

Antiviral and mechanistic studies of IFN-α treatment of HBV infection have been performed in vitro, in transgenic and immunodeficient mouse models, and in peripheral blood from CHB patients, but there is very little data regarding the intrahepatic response to IFN-α treatment in an immunocompetent host. A baseline (i.e. pre-treatment) intrahepatic transcriptional signature of response to treatment with pegylated IFN-α and adefovir (response defined as HBeAg loss, HBV DNA <2,000 IU/mL and ALT normalization) has recently been described [20]. However, due to the difficulty in obtaining multiple liver biopsy specimens from chronically infected HBV patients, longitudinal evaluation of the intrahepatic response to IFN-α treatment is only possible with an animal model. Since ethical and cost considerations limit the use of chimpanzees for biomedical research and there is no small animal model of natural HBV infection, we selected the woodchuck model for this purpose.

The Eastern woodchuck (*Marmota monax*) is naturally infected with WHV, a hepadnavirus which is genetically closely related to human HBV [21]. WHV infection displays a disease course similar to that in HBV-infected persons [21]. Although the woodchuck model has been used in a number of studies to characterize antiviral response to IFN-α treatment [22,23] these studies relied on adenovirus delivery of woodchuck IFN-α or utilized a recombinant human hybrid (B/D) IFN-α. Furthermore, these studies did not define the molecular basis of antiviral response. We recently described the sequencing, assembly and annotation of the woodchuck transcriptome, together with the generation of custom woodchuck microarrays. Using this new platform, we established the translational value of the woodchuck model and characterized the immune determinants of WHV clearance during self-limiting infection [24,25]. Since these studies yielded important insights into immune responses in the liver during hepadnavirus infection, in the current study we used a similar approach to characterize the intrahepatic transcriptional signature associated with antiviral response to recombinant woodchuck IFN-α treatment.

## Results

### Single dose IFN-α study in WHV-negative woodchucks

The amino acid sequence and in vitro antiviral activity of woodchuck IFN-α5 (wIFN-α) have previously been described [26,27]. wIFN-α was expressed, purified and biological activity confirmed as described in the Methods. The tolerability and pharmacodynamic activity of wIFN-α were then evaluated in a single dose study in WHV-negative woodchucks. Subcutaneous administration of a single dose of 2, 20 or 200 μg wIFN-α per animal (n = 3/group), induced dose-dependent increases in ISG and cytokine mRNA expression in the blood relative to the placebo group (S1 Fig). Pharmacokinetic (PK) analysis of serum wIFN-α levels was not performed due to the lack of a sufficiently sensitive quantitative method (see Methods). There was a trend towards changes in several hematological and clinical chemistry parameters at the higher doses, although these were likely due to the drawing of large blood volumes over a short time period.

### IFN-α efficacy study in chronic WHV carrier woodchucks

**Fig 1:**
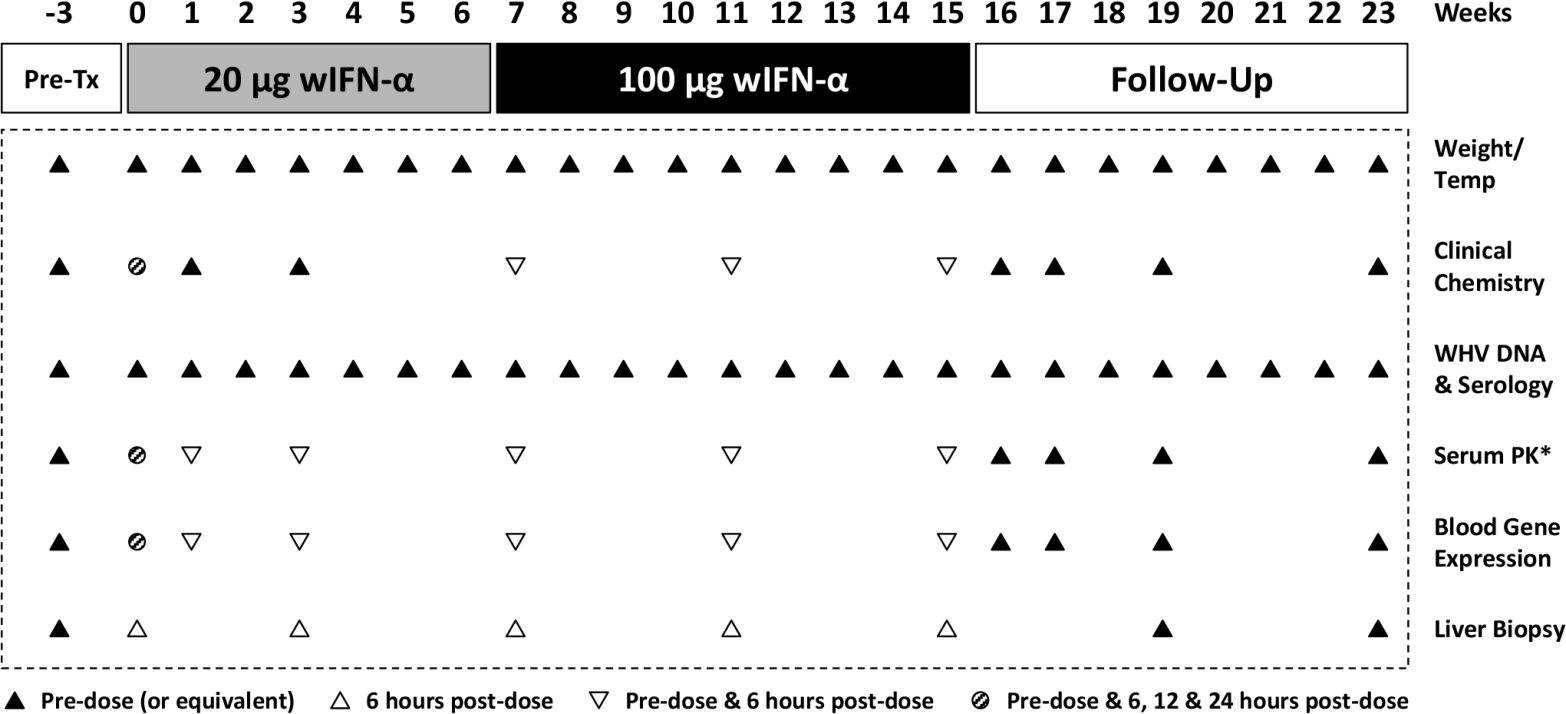
Design of the wIFN-α treatment study in woodchucks chronically infected with WHV. Chronic WHV carrier woodchucks were dosed three times a week (TIW) for 15 weeks with placebo, or for 7 weeks with 20 μg wIFN-α followed by another 8 weeks with 100 μg wIFN-α (15 weeks total). Animals were typically followed for additional 8 weeks after the treatment period (follow-up), although this was extended by two weeks for two wIFN-treated woodchucks (animals M1002 and M1004) that had no evidence of viral recrudescence at the end-of-study (week 23). *PK analysis of serum wIFN-α levels was not performed due to the lack of a sufficiently sensitive quantitative method (see Methods).

**Fig 2:**
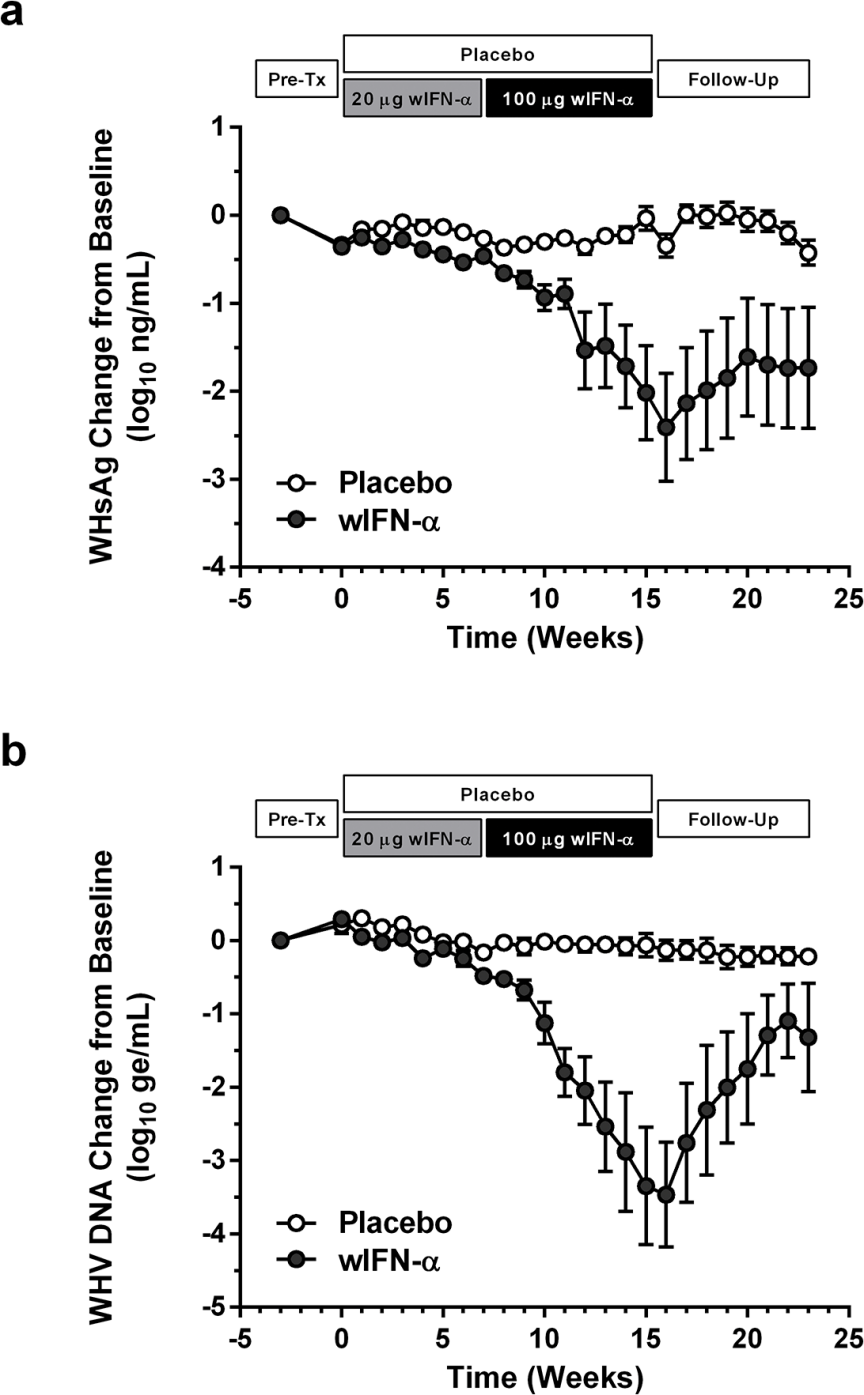
wIFN-α treatment of chronic WHV carriers induces suppression of serum antigenemia and viremia. Change in serum (a) WHsAg and (b) WHV DNA relative to week -3 (pre-treatment baseline). Circles indicate the mean of each group (open: placebo, closed: wIFN-α), and the error bars represent the standard error of the mean. The WHsAg level for two wIFN-treated animals was ≤ lower limit of detection (LLOD; 20 ng/mL) at various times during the study (S2 Fig); the LLOD was used to estimate the WHsAg decline at these timepoints. Note that seven animals (three in the placebo group, four in the wIFN-α group) died during the study, and one animal in the wIFN-α group (M1004) was excluded from the analysis since it was likely naturally clearing WHV as the study initiated (see Table 1).

**Fig 3:**
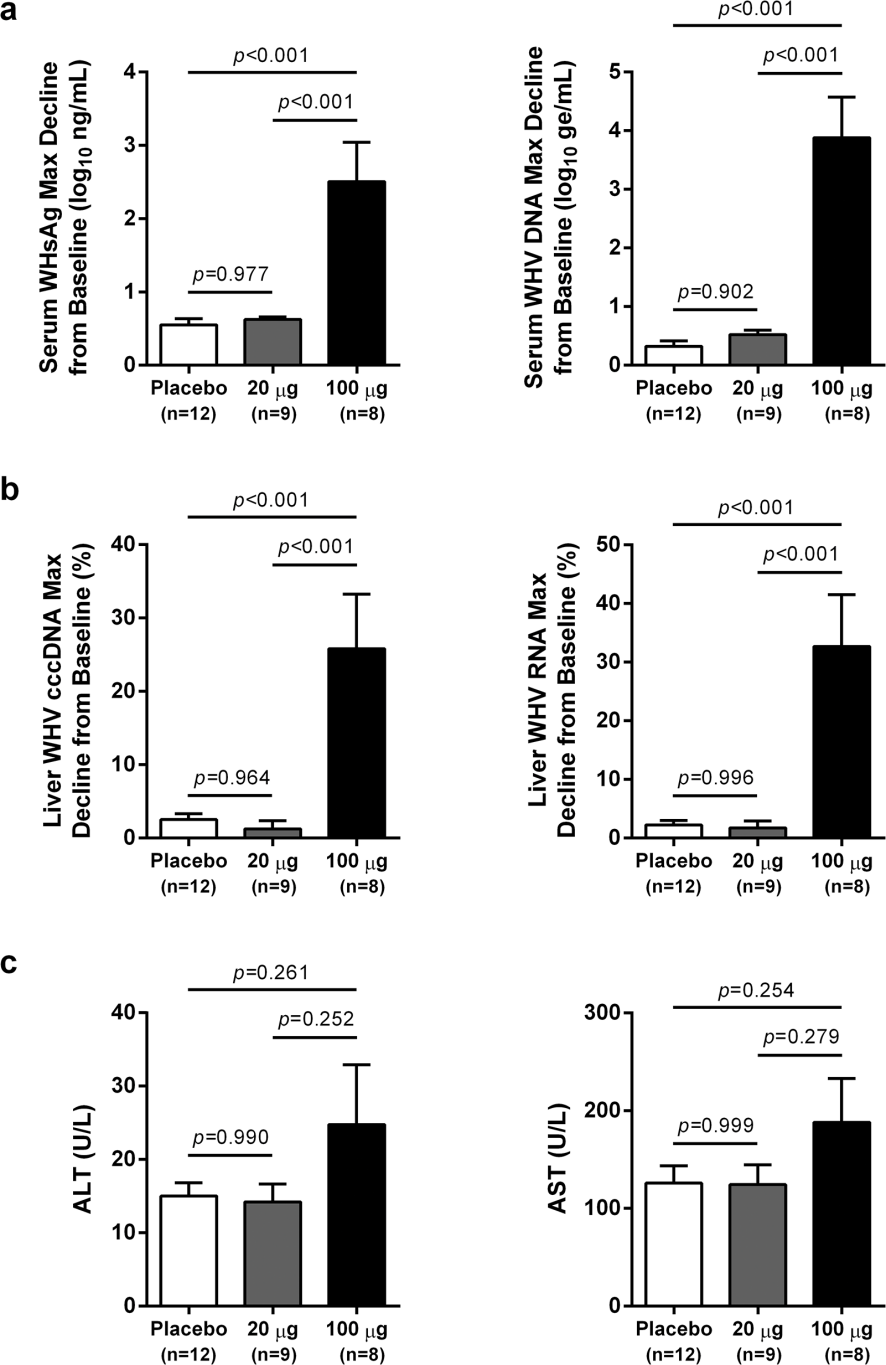
High dose wIFN-α significantly inhibits WHV. Maximum reductions in (a) serum and (b) intrahepatic viral parameters, and (c) maximum serum ALT and AST levels in response to placebo, low dose (20 μg) wIFN-α and high dose (100 μg) wIFN-α treatment. Changes in viral parameters were calculated relative to week -3 (pre-treatment baseline), with the exception of F1018 (wIFN-α group), for which week 0 was used as the baseline for the intrahepatic cccDNA and RNA analyses. The bar height indicates the mean of each group, and the errors bars represent the standard error of the mean. The lowest WHsAg level for two wIFN-treated animals (M1002 and F1022) was ≤ LLOD (20 ng/mL), and so the LLOD was used to estimate the maximum WHsAg decline. Per the sampling scheme outlined in Fig 1, the following data was included in the analyses: maximum reduction in serum WHV DNA and WHsAg at weeks 0–16 (placebo), 0–7 (20 μg dose) and weeks 8–16 (100 μg dose); maximum reduction in intrahepatic cccDNA and RNA at weeks 0–15 (placebo), weeks 0 and 3 (20 μg dose) and weeks 7, 11 and 15 (100 μg dose); maximum serum ALT and AST levels at weeks 0–16 (placebo), 0–7 (20 μg dose) and weeks 11–16 (100 μg dose). Animals in the wIFN-α group were only included if data from all relevant time-points was available, with the exception of F1020, for which no week 16 sample was available for serum WHV DNA, WHsAg, ALT and AST analysis. Statistical significance was calculated by one-way ANOVA with Tukey's multiple comparison correction.

The antiviral efficacy of wIFN-α was then evaluated in a repeat-dose study in adult woodchucks chronically infected with WHV. To model vertical transmission in humans, chronic infection in these animals was established by neonatal WHV infection. The study design is described in Fig 1. To match the frequency of non-pegylated IFN-α dosing in CHB patients, animals (n = 12/group) were dosed subcutaneously three times per week (TIW) on Monday, Wednesday and Friday with either placebo (vehicle control) or wIFN-α for a total of 15 weeks. Based on activity and safety considerations from the single dose study in WHV-negative woodchucks, the 20 μg dose was selected as the starting dose for the efficacy study. Initially wIFN-α was given for 7 weeks at a low dose of 20 μg/animal TIW. However, since an interim analysis indicated that this dose did not induce a significant decline in serum WHsAg or WHV DNA (Figs 2 and 3A), at the start of week 7 the wIFN-α dose was increased to 100 μg/animal TIW. Thus, in the wIFN-α treatment group, animals received a low dose of wIFN-α for 7 weeks (21 doses total), followed by a high dose of 100 μg/animal for another 8 weeks (24 doses total). Note that one animal in this group (M1004) was excluded from the analyses described below since it was likely naturally clearing WHV as the study initiated (Table 1).

**Table 1:** Serum and liver WHV measurements in the wIFN-α and placebo groups.

Treatment group	Woodchuck ID#	Response group^a^	Baseline serum WHV DNA (log_10_ ge/mL)^b^	Baseline serum WHsAg (log_10_ ng/mL)^b^	Max decline in serum WHV DNA (log_10_ ge/mL)^c^	Max decline in serum WHsAg (log_10_ ng/mL)^c^	Max decline in liver cccDNA (%)^c^	Max decline in liver WHV DNA (%)^c^	Max decline in liver WHV RNA (%)^c^
wIFN-α	M1002	R	9.97	5.55	4.87	4.25^d^	55	62	60
	M1003	PR	10.58	5.55	4.26	1.54	22	33	31
	M1004	N/A^e^	9.40	4.82	5.70	3.52^d^	68	85	81
	M1006	N/A^†^	10.91	5.62	-0.16	0.44	1	-3	0
	M1007	N/A^†^	10.62	5.92	0.98	0.76	5	9	6
	M1012	NR	10.60	5.66	1.21	0.66	4	15	10
	F1013	R	10.66	5.85	3.82	3.55	21	25	20
	F1014	NR	10.60	5.87	0.84	0.70	4	9	6
	F1018	PR	10.98	5.84	4.22	1.85	22	32	29
	F1020	N/A^†^	11.20	5.89	6.44	3.14	55	80	71
	F1022	R	10.96	5.67	6.13	4.37^d^	47	72	56
	F1023	N/A^†^	11.41	5.71	0.16	0.28	0	3	0
Placebo	M1001	N/A	10.90	5.19	0.06	0.14	3	2	-3
	M1005	N/A	10.38	5.43	0.04	0.53	4	4	3
	M1008	N/A	10.62	5.49	0.82	0.80	6	10	5
	M1009	N/A	10.86	5.68	0.34	0.44	4	3	3
	M1010	N/A^†^	11.57	5.75	0.65	0.37	3	4	4
	M1011	N/A	11.26	5.92	0.35	0.76	5	3	6
	F1015	N/A	10.59	5.63	0.06	0.80	2	2	1
	F1016	N/A	10.15	5.41	0.34	0.87	4	5	1
	F1017	N/A^†^	10.37	5.51	0.45	0.93	1	2	3
	F1019	N/A	11.39	5.94	1.07	0.91	8	12	7
	F1021	N/A	10.81	4.82	0.71	0.04	6	8	2
	F1024	N/A^†^	10.63	5.60	-0.15	0.47	1	-2	-2

N/A: not applicable.

^a^Treatment response groups were defined as follows: R, responder ≥1 log_10_ reduction in WHsAg at week 15 (end-of-treatment) and week 23 (end-of study); PR, partial responder ≥1 log_10_ reduction in WHsAg at week 15 but not week 23; NR, non-responder <1 log_10_ reduction in WHsAg at week 15 and week 23.

^b^Serum WHV DNA and WHsAg levels at week -3 (pre-treatment baseline).

^c^The serum and intrahepatic viral endpoint differentials at weeks 0–23/25 (end-of-study) were calculated relative to the week -3 (pre-treatment) timepoint.

^d^WHsAg levels for animals M1002, M1004 and F1022 were ≤ lower limit of detection (LLOD; 20 ng/mL) at one or more timepoints; the LLOD was used to calculate the maximum WHsAg decline for these animals.

^e^M1004 had substantially lower week-3 baseline WHsAg and WHV DNA relative to the other study animals; WHsAg levels in this animal dropped by 1.5 log_10_ between week -3 and the start of treatment (week 0), and then fell to ≤ LLOD after only 2 weeks of low dose wIFN-α. This animal subsequently developed very high titer anti-WHs (S1 Table).

†Animal died during the study: M1006 (wIFN-α group); euthanized week 3 due to deteriorating health conditions which were likely related to underlying metabolism abnormalities, M1007 (wIFN-α group); died week 11, likely due to biopsy-related hemorrhage, F1020 (wIFN-α group); died week 16, cause of death unknown, F1023 (wIFN-α group); died week 2, likely related to severe pneumonia, M1010 (placebo group); euthanized week 17 due to symptoms associated with terminal HCC, F1017: (placebo group); euthanized week 17 due to symptoms associated with terminal HCC, F1024 (placebo group); died week 11, likely due to biopsy-related hemorrhage.

### IFN-α treatment of chronic WHV carriers induced variable suppression of serum antigenemia and viremia

In contrast to low dose (20 μg) wIFN-α, high dose (100 μg) wIFN-α treatment induced a rapid decline in serum WHsAg and WHV DNA (Figs 2 and S2), which was statistically significant relative to the placebo group (Fig 3A). The maximum reduction of serum WHsAg and WHV DNA was at week 16 in most animals, with a mean maximal reduction of 2.0 log_10_ for WHsAg and 3.0 log_10_ for viral load. Notably, wIFN-α treatment induced the complete loss of detectable (<20 ng/mL) WHsAg in one animal (F1022), although WHV DNA was still detectable (>1,000 genome equivalents (ge)/mL) at all time-points (S2 Fig). After completion of treatment there was WHsAg and WHV DNA rebound in most woodchucks, albeit not always to pre-treatment levels (Figs 2 and S2). There was a high degree of variability in the antiviral response of individual woodchucks in regard to the kinetics and magnitude of serum WHsAg and WHV DNA decline, as well to the time interval between cessation of treatment and return of these viral parameters to pre-treatment levels (S2 Fig). For correlative analyses with treatment response (see below), response groups were defined as the following: R, responder ≥1 log_10_ reduction in WHsAg at week 15 (end-of-treatment) and week 23 (end-of study) (n = 3 animals); PR, partial responder ≥1 log_10_ reduction in WHsAg at week 15 but not week 23 (n = 2 animals); NR, non-responder <1 log_10_ reduction in WHsAg at week 15 and week 23 (n = 2 animals) (Table 1). Notably, baseline (pre-treatment) levels of serum WHsAg and WHV DNA were comparable in these different treatment response groups (Table 1). The four animals in the wIFN-treatment group that did not survive until end-of study (see below), together with animal M1004 which was likely naturally clearing infection, were excluded from treatment response analyses (Table 1).

### IFN-α treatment significantly reduced the hepatic levels of WHV nucleic acids, but induced anti-WHs antibodies in only two animals

High dose wIFN-α treatment significantly reduced intrahepatic cccDNA, WHV DNA replicative intermediate (RI) and WHV RNA levels (Figs 3B and S3). Reductions in these intrahepatic parameters typically correlated with reductions in serum WHsAg and viral load (Table 1). Only two woodchucks (M1004 and F1022) with sustained WHsAg reduction developed consistently detectable anti-WHs antibodies (S1 Table), one of which (M1004) was likely naturally clearing WHV as the study initiated (Table 1). The overall seroconversion rate was therefore 0/9 (placebo group) and 1/7 (wIFN-α group) for animals that survived until end-of-study (excluding M1004).

### Tolerability of IFN-α treatment in chronic carrier woodchucks

**Fig 4:**
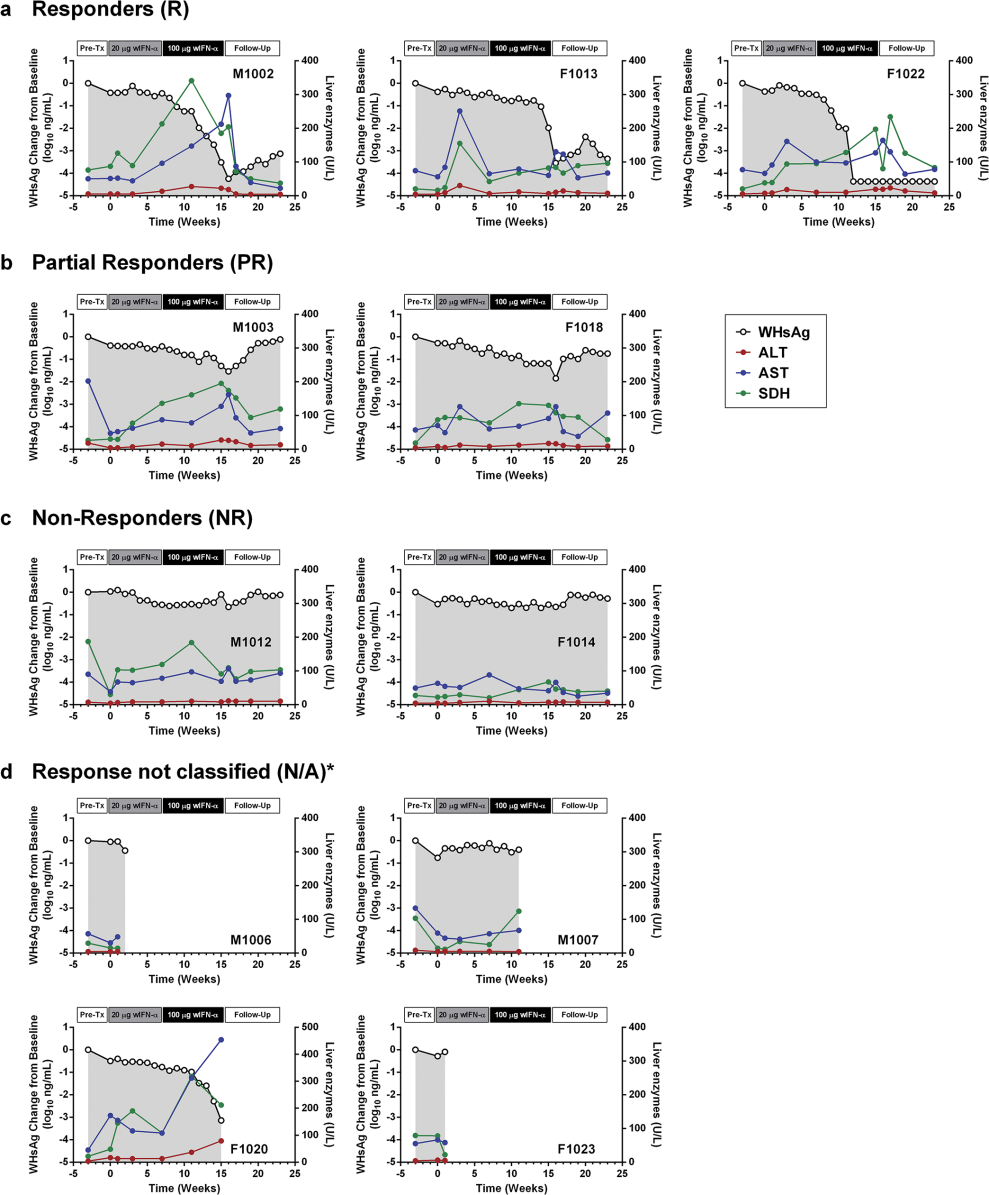
Serum WHsAg and liver enzymes for individual wIFN-treated animals. Serum WHsAg (black open circles) is plotted on the left y-axis. Serum ALT (red circles), AST (blue circles) and SDH (green circles) are all plotted on the right y-axis. The treatment response group classifications (a-d) are described in Table 1. *Animals died prior to end-of-study. Note all data was from pre-dose (or equivalent).

wIFN-α treatment was well-tolerated, and there were no signs of overt toxicity based on gross observations, body weights, hematology or clinical chemistry. Although several animals died during treatment, the causes of death (e.g. HCC-related conditions, biopsy complications) were likely not treatment related (Table 1). There was a trend towards elevated serum ALT and AST levels during high dose treatment, but on a group level these overall differences were not statistically significant (Fig 3C). This is reflected in a poor temporal association between peak antiviral response and elevation of ALT, AST and SDH in some animals (Fig 4). Similarly, even though there was considerable fluctuation in liver histology scores in both placebo and wIFN-α groups (S1 Table), antiviral response was correlated temporally with an increase in liver inflammation in some (although not all) wIFN-treated animals (S4 Fig). Conversely, baseline liver enzyme levels and pre-treatment histology scores were comparable in the different treatment response groups (Figs 4 and S4).

### IFN-α treatment induced expression of ISGs and T_H_1-type cytokines in the blood of chronic WHV carriers

**Fig 5:**
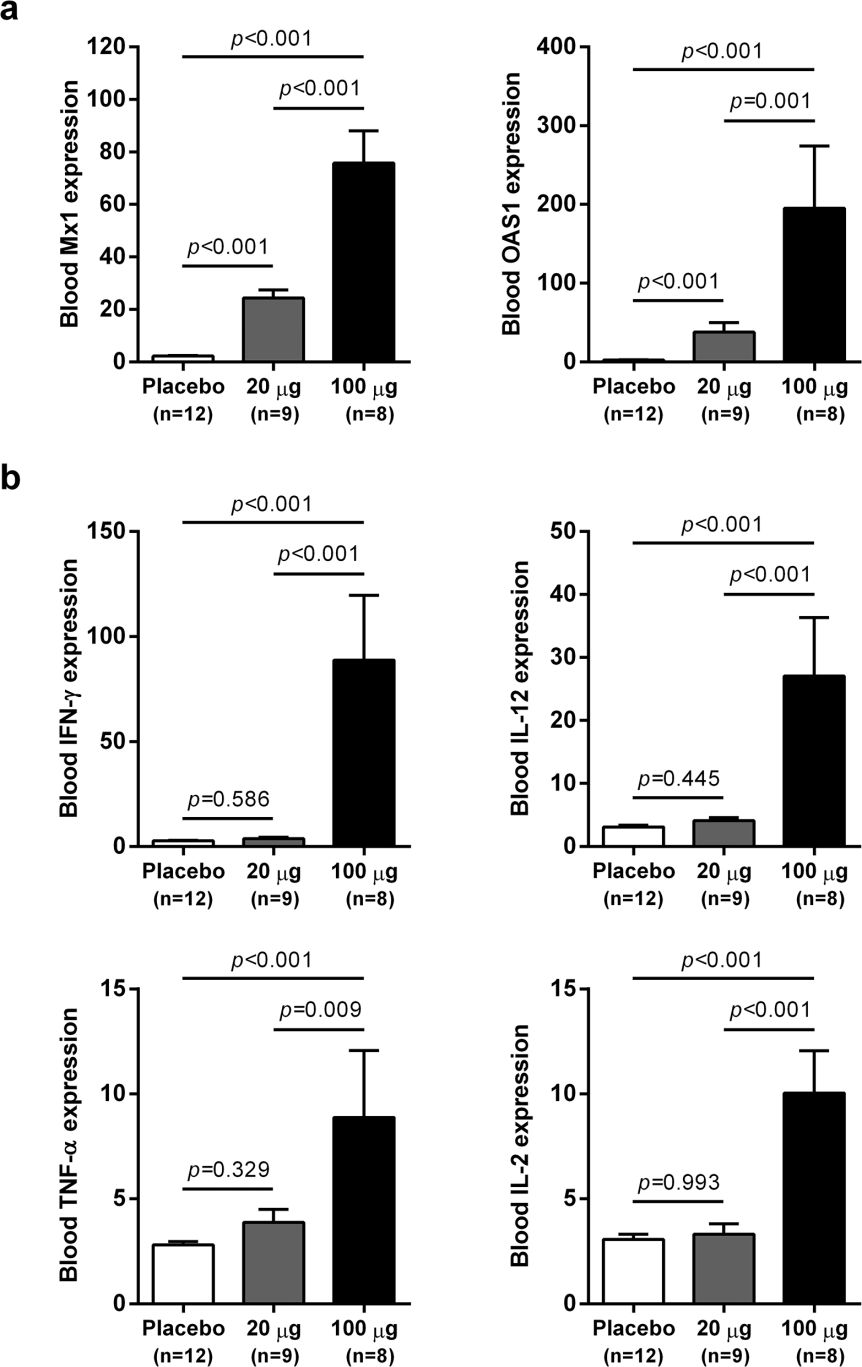
Differential induction of whole blood gene expression by low dose and high dose wIFN-α. qRT-PCR data for (a) ISGs and (b) T_H_1-type cytokine genes expressed as fold-change relative to week 0 pre-dose (pre-treatment baseline). The bar height indicates the mean maximal fold-change for each group, and the errors bars represent the standard error of the mean. Placebo: maximal induction at 6 hours post-dose at weeks 0, 1, 3, 7, 11 and 15. 20 μg: maximal induction at 6 hours post-dose at weeks 0, 1 and 3. 100 μg: maximal induction at 6 hours post-dose at weeks 7, 11 and 15. Animals from the wIFN-α treatment group were only included if data from all relevant time-points was available. Statistical significance was calculated with log-transformed values by one-way ANOVA with Tukey's multiple comparison correction.

wIFN-α treatment induced dose-dependent increases in blood ISG mRNA expression. There was significant induction at both low and high dose levels, with a larger increase observed for the higher dose (Fig 5A). In contrast, only high dose treatment significantly induced the expression of various T helper cell type 1 (T_H_1)-type cytokines (Fig 5B). Given that only high dose treatment was associated with a significant antiviral response, this suggests cellular immunity (and associated cytokines) may play a role in and/or be a useful biomarker of treatment response. Although comparative analysis is limited by small animal numbers in each response group, a role for cellular immunity in antiviral response is also suggested by the significant difference in IFN-γ expression in animals with an on-treatment response (R and PR) relative to those with no treatment response (NR) (S5 Fig).

### IFN-α treatment substantially altered intrahepatic gene expression in chronic WHV carrier woodchucks

**Fig 6:**
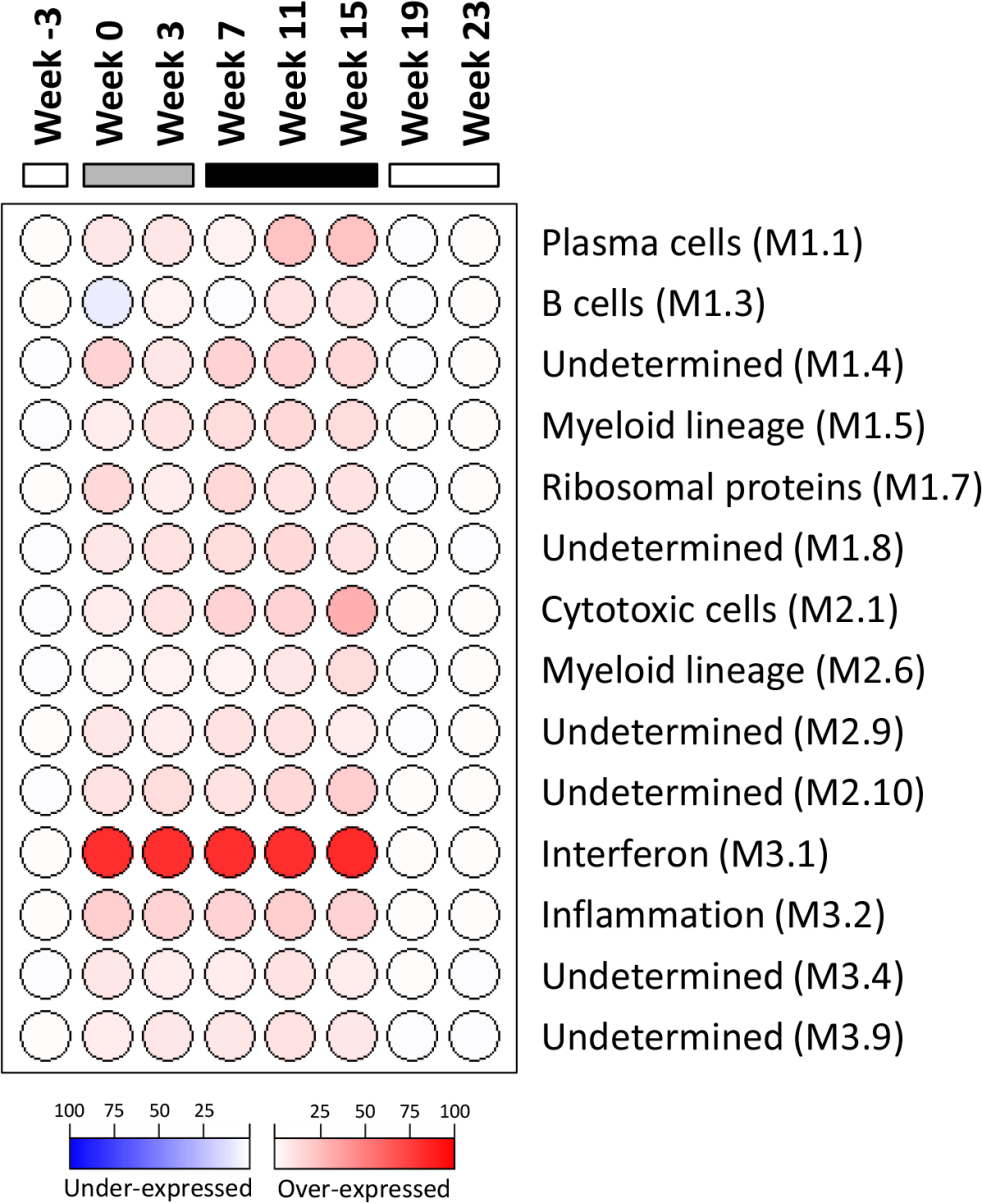
Modular analysis of intrahepatic transcriptional signatures in the wIFN-α treatment group. Data from all available wIFN-treated animals (n = 5–11) were included at each time-point. Spot intensity (red: over-expressed; blue: under-expressed) denotes the percentage of transcripts significantly changed in each module (M) and is defined by the scale bar. The functional interpretation of each module [29] is displayed on the right. Only modules with enrichment greater than 10% at one or more time-point are displayed. At each time-point, all genes selected for modular analysis had an absolute fold-change > 1.5 with a Benjamini-Hochberg corrected *FDR*<0.05 relative to the time-matched placebo group. The horizontal bars together with the week numerators indicate the study stage, as described in Fig 1.

As outlined in Fig 1, intrahepatic transcriptional profiles of placebo-treated and wIFN-treated animals were determined by RNA-Seq at various times during the study. RNA-Seq was performed rather than using the microarray platform from previous studies [24,25] because this method has superior concordance with qRT-PCR data [28] and also enabled generation of a more complete (version 2) woodchuck transcriptome assembly (S2 Table). Principal Component Analysis (PCA) demonstrated that wIFN-α treatment substantially altered gene expression within the liver of chronic carrier animals (S6 Fig). In contrast to the significant difference in antiviral response, there were only relatively modest differences (restricted to PC#2) between intrahepatic transcriptional changes induced by low dose (20 μg) and high dose (100 μg) wIFN-α treatment. A gene module approach [29] confirmed that there was substantial modulation of intrahepatic gene expression by wIFN-α overall, with only moderate differences between low and high dose treatment (Fig 6). The modular signature for wIFN-α treatment revealed an increase (>10% of the transcripts in each module significantly up-regulated) in the number of differentially expressed genes in the IFN response (Module, M3.1), cytotoxic cell (NK cell/CD8^+^ T cell) (M2.1), plasma cell (M1.1), B cell (M1.3), myeloid cell lineage (M1.5 and M2.6) and inflammation (M3.2) modules (Fig 6). Consistent with an increase in liver inflammation in many wIFN-treated animals (S4 Fig), the transcriptional data suggest that wIFN-α induced migration of immune cells into the liver and/or proliferation of intrahepatic immune cells.

### Intrahepatic expression of the majority of antiviral ISGs did not correlate with the antiviral response to IFN-α treatment

**Fig 7:**
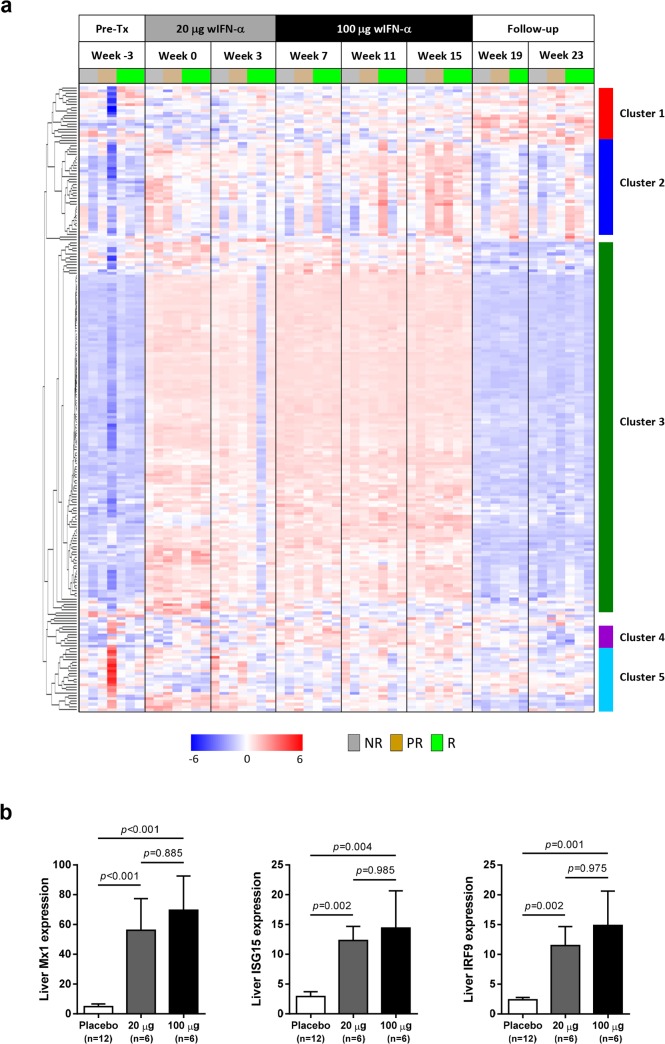
Comparable induction of intrahepatic expression of most ISGs with low dose and high dose wIFN-α. (a) Unsupervised hierarchical clustering of differentially expressed intrahepatic ISGs of animals that were responders (R, n = 3), partial responders (PR, n = 2) or non-responders (NR, n = 2) to wIFN-α treatment. Note that there was no week 19 sample for the responder group animal M1002 and the week 25 sample (end-of-study for this responder animal) was included at week 23 (end-of-study for most animals) for ease of data comparison. The sources of the ISGs are described in S3 Table. Heatmap columns represent samples from individual animals collected at the indicated times, and rows represent different genes (n = 209). Red and blue coloring of cells represents high and low expression levels (normalized count data), respectively, as indicated by the scale bar for log_2_ normalized values. (b) qRT-PCR data expressed as fold-change relative to week -3 (pre-treatment baseline). The bar height indicates the mean of each group, and the errors bars represent the standard error of the mean. Placebo: maximum induction at 6 hours post-dose at weeks 0 and 7. 20 μg: sample collected 6 hours post-first dose of 20 μg wIFN-α (week 0). 100 μg: sample collected 6 hours post-first dose of 100 μg wIFN-α (week 7). Animals from the wIFN-α treatment group were only included if both 20 μg wIFN-α (week 0) and 100 μg wIFN-α (week 7) data was available (see S4 Table). Statistical significance was calculated with log-transformed values by one-way ANOVA with Tukey's multiple comparison correction.

In contrast to the differential antiviral response (Fig 3A and 3B) and dose-dependent ISG induction in the periphery (Fig 5A), module analysis revealed a striking increase (>80% of the transcripts significantly up-regulated) in the intrahepatic IFN response module (M3.1) at all on-treatment time-points, regardless of wIFN-α dose (Fig 6). Consistent with the modular analysis, low dose and high dose wIFN-α treatment were both associated with strong induction of a large number of intrahepatic ISGs, including many antiviral effector genes (Fig 7A, cluster 3). Furthermore, there was no apparent difference between the intrahepatic expression of these ISGs in animals with a treatment response (R and PR) and those with no treatment response (NR). Comparable induction of select ISGs in the liver by low and high dose wIFN-α treatment (regardless of treatment response) was confirmed by qRT-PCR (Fig 7B, S4 Table). Taken together, these data indicate that the antiviral response to wIFN-α does not correlate with the intrahepatic expression of the majority of ISGs, suggesting they do not play a key role in the antiviral response to treatment (see Discussion). Furthermore, pre-treatment (week -3) ISG levels were comparable in the different response groups (Fig 7A), indicating that baseline ISG expression was not an important determinant of treatment response.

In the context of defining the molecular basis of IFN-α treatment response, the APOBEC proteins are ISGs of particular interest since various family members have been reported to be restriction factors for HBV [13]. It is therefore notable that the intrahepatic expression profile of *APOBEC3H* (A3H) was unlike the majority of antiviral ISGs, in that it was selectively induced by high dose wIFN-α treatment (Table 2). However, the degree of *A3H* induction was modest (maximum 3.6-fold) relative to many other ISGs, consistent with low *A3H* induction by IFN-α in purified primary human hepatocytes [13]. Furthermore, intrahepatic induction of *A3H* was only statistically significant at end-of-treatment (week 15), suggesting that it is not likely to be a main mediator of the wIFN-α antiviral response. In contrast to *A3H*, *A3D* and *A3F* were not significantly modulated (FDR<0.05, FC>2) by wIFN-α treatment. Other APOBEC3 family members (including A3A) were not available in the woodchuck transcriptome assembly.

**Table 2:** High dose wIFN-α significantly induced intrahepatic expression of T cell, NK cell and IFN-γ response genes.

Gene^a^ ^,^ ^b^	Pre-Tx	20 μg wIFN-α		100 μg wIFN-α			Follow-Up	
	W-3 (n = 11)	W0 (n = 11)	W3 (n = 9)	W7 (n = 9)	W11 (n = 8)	W15 (n = 8)	W19 (n = 6)	W23 (n = 5)
PLA2G2A	-2.10	1.55	-1.81	2.70	7.30^c^	11.23	1.06	-4.80
CXCL9	-1.29	1.85	2.21	4.51	11.04	10.35	-1.04	-1.73
PDCD1	-1.07	-1.10	1.13	1.39	5.15	6.86	1.22	-1.76
APOBEC3H	-1.00	1.13	1.25	1.51	2.49	3.58	1.23	-2.15
TBX21	1.20	1.70	1.72	1.84	3.10	3.33	-1.30	-2.27
KLRK1	1.48	1.35	1.52	1.80	2.54	2.99	-1.25	-2.18
CD3D	1.12	1.10	1.32	1.31	2.27	2.39	-1.12	-1.53
FOXP3	1.10	-1.12	-1.14	1.09	1.81	2.29	1.18	-1.57
CD8A	-1.01	-1.12	-1.20	-1.05	1.68	1.88	-1.11	-1.20

RNA-Seq data for genes selectively induced by high dose (100 μg) but not by low dose (20 μg) wIFN-α. The full list of genes induced by high dose wIFN-α only (n = 468 genes) is displayed in S8C Table.

^a^Fold-change ratio values relative to time-matched placebo animals (n = 5–12) are displayed, with underlining denoting statistical significance (FDR<0.05). For genes where wIFN-treated > placebo, the fold change ratio was calculated by 1 x (treated/placebo), i.e. 1.50 equals a 50% increase in treated vs. placebo. For genes where wIFN-treated < placebo, the fold change ratio was calculated by -1 x (placebo/ treated), i.e. -1.50 equals a 50% decrease in treated vs. placebo.

^b^Hugo symbols are used for gene names. Alternative gene names; *CXCL9*: *MIG*, *PDCD1*: *PD-1*, *TBX21*: *T-bet*, *KLRK1*: *NKG2D*.

^c^Fold-change ratio for *PLA2G2A* at week 11 was close to statistical significance (FDR = 0.063).

W: week.

### Intrahepatic NK and T cell transcriptional signatures correlate with the antiviral response to IFN-α treatment

**Fig 8:**
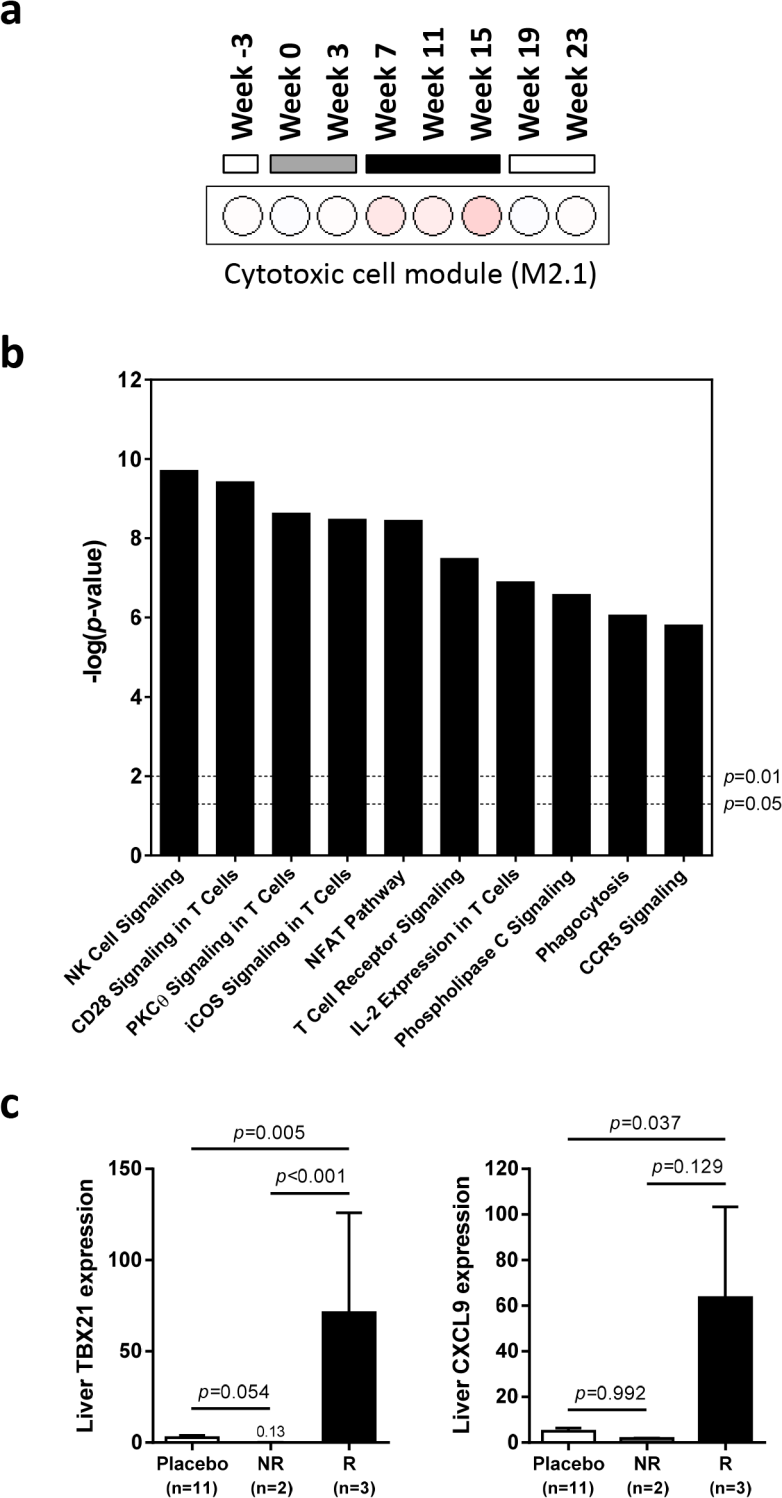
Characterization of intrahepatic transcriptional signature associated with response to wIFN-α treatment. Analysis of genes (n = 468) differentially induced by high dose (100 μg) wIFN-α (see S7 Fig, “High dose”). (a) Modular analysis of intrahepatic gene expression, as described in Fig 6. Only the cytotoxic cell module (M2.1) had enrichment greater than 10% at one or more time-point. (b) Top canonical pathways identified by Ingenuity Pathway Analysis. Pathway enrichment was calculated with the Fisher’s exact test with multiple testing correction by the Benjamini and Hochberg method. The–log(*p*-value) for *p* = 0.05 and *p* = 0.01 significance levels are indicated. (c) qRT-PCR data expressed as fold-change relative to week -3 (pre-treatment baseline). The bar height indicates the mean of each group, and the errors bars represent the standard error of the mean. Placebo: mean induction at 6 hours post-dose at weeks 0, 7 and 15 in placebo-treated animals. wIFN-α: induction at 6 hours post-dose at week 15 in non-responder (NR, n = 2) or responder (R, n = 3) animals. No samples from partial responder (PR) animals were available for qRT-PCR analysis (S6 Table). Statistical significance was calculated with log-transformed values by one-way ANOVA with Tukey's multiple comparison correction.

Since there was a strong association between wIFN-α dose and antiviral response (Fig 3A and 3B), we reasoned that determining which genes were selectively induced by high dose wIFN-α would enable the identification of genes and/or pathways closely associated with treatment response. This approach identified genes that were selectively modulated during high dose wIFN-α treatment (S7 Fig, high dose n = 468), as well as genes induced only by low dose treatment (low dose n = 29) or by both low and high dose wIFN-α (low & high dose n = 775). The full gene list from each set is displayed in S8 Table. Consistent with the previous analyses, module analysis (M3.1) and Ingenuity Pathway Analysis (IPA) confirmed significant induction of an IFN-α response at both low dose and high dose wIFN-α treatment (S8 Fig). In contrast, module analysis revealed that cytotoxic cell (NK cell/CD8^+^ T cell) responses were selectively induced by high dose wIFN-α treatment, and hence were temporally associated with treatment response (Fig 8A). Significant enrichment of NK and T cell signatures with high dose wIFN-α treatment was confirmed by IPA (Fig 8B). To complement the approach focused on identifying genes selectively induced by high dose wIFN-α, Weighted gene coexpression network analysis (WGCNA) was used to identify modules of co-regulated treatment-induced genes that correlated most closely with antiviral response (S5 Table, Modules 1 and 2). These modules were also significantly enriched for NK and T cell associated genes (S9 Fig), consistent with the trend for induction of an NK/T cell signature in animals that had an antiviral response to treatment (M2.1, S10 Fig). Notably, these diverse analytical approaches identified common intrahepatic transcriptional signatures associated with treatment response, suggesting that NK/T cells play an important role in the antiviral response to wIFN-α treatment.

On the individual gene level, induction of T cell associated genes (*CD3D*, *CD8A*) suggests that there is migration of T cells into the liver and/or proliferation of intrahepatic T cells during high dose wIFN-α treatment (Table 2). Expression of the T cell T_H_1-type transcription factor *T-bet* (*TBX21*) was also significantly induced during high dose treatment (Table 2). Strikingly, qRT-PCR analysis revealed that *T-bet* expression was strongly induced by high dose treatment in animals with treatment response but not in animals without an antiviral response (Fig 8C and S6 Table). This is notable since it may indicate improved functionality (antigen-specific proliferation and IFN-γ production) of intrahepatic HBV-specific CD8^+^ T cells, particularly since high dose wIFN-α also induced IL-12 expression (Fig 5B) [30]. However, it is important to note that this transcriptional analysis cannot determine whether *T-bet* is expressed by virus-specific or virus non-specific CD8^+^ T cells, or potentially other cell types [31]. Induction of *NKG2D* (*KLRK1*; activating receptor) expression, but not *NKG2A* (*KLRC1*; inhibitory receptor), *CD16* (*FCGR3A*) or *CD56* (*NCAM1*) (Tables 2 and 3), is consistent with activation, but not migration or proliferation of intrahepatic NK cells.

**Table 3:** Intrahepatic expression of genes induced by both low and high dose wIFN-α or induced by neither.

Gene^a^ ^,^ ^b^	Pre-Tx	20 μg wIFN-α		100 μg wIFN-α			Follow-Up	
	W-3 (n = 11)	W0 (n = 11)	W3 (n = 9)	W7 (n = 9)	W11 (n = 8)	W15 (n = 8)	W19 (n = 6)	W23 (n = 5)
USP18	2.67	21.90	48.23	43.81	44.72	49.74	1.29	-1.25
IDO1	1.28	14.29	6.42	10.47	20.83	24.01	1.01	-2.10
CD274	1.32	4.64	2.88	4.98	5.50	5.49	-1.09	-1.59
TNFSF10	1.10	3.87	4.84	5.46	5.61	5.46	-1.08	-1.07
FASLG	1.11	2.29	2.01^c^	2.05	2.98	4.38	-1.47	-2.31
PRF1	1.06	1.86	2.66	2.74	5.01^c^	4.02	-1.01	-1.30
FAS	1.21	3.05	2.55	3.35	2.96	3.09	-1.05	-1.08
SOCS1	1.04	2.68	1.82	2.18	2.25	2.63	1.06	1.20
SOCS3	1.49	2.30	1.85	2.07	1.47	1.80	-1.09	-1.34
KLRC1	1.10	1.31	1.82	1.93	1.78	1.75	-1.68	-4.01
FCGR3A	-1.02	-1.04	-1.31	-1.16	1.32	1.73	-1.38	-1.70
TGFB1	1.10	-1.26	-1.20	-1.10	1.23	1.36	-1.29	-1.37
IL10	1.03	1.05	1.02	1.03	-1.00	-1.01	-1.11	-1.28
NCAM1	2.27	3.58	-1.43	-3.13	1.57	-2.62	-2.65	-1.04

RNA-Seq data for select genes significantly induced by both low dose (20 μg) and high dose (100 μg) wIFN-α (top 9 rows), or induced by neither (bottom 5 rows). The full list of genes induced by both low dose and high dose wIFN-α (n = 775 genes) is displayed in S8B Table.

^a^Fold-change ratio values relative to time-matched placebo animals (n = 5–12) are displayed, with underlining denoting statistical significance (FDR<0.05). Fold-change ratio was calculated as described for Table 2.

^b^Hugo symbols are used for gene names. Alternative gene names; *CD274*: *PD-L1*, *TNFSF10*: *TRAIL*, *KLRC1*: *NKG2A*, *FCGR3A*: *CD16*, *NCAM1*: *CD56*.

^c^Fold-change ratios for *FASLG* at week 3 and *PRF1* at week 11 were close to statistical significance (FDR = 0.097 and FDR = 0.062, respectively).

W: week.

### Intrahepatic NK and/or T cells likely inhibit WHV via both cytolytic and non-cytolytic mechanisms during IFN-α treatment

As discussed previously, the peak antiviral response to treatment and elevation of liver injury biomarkers were temporally correlated in some animals (Fig 4), indicating that wIFN-α induced killing of WHV-infected hepatocytes. This biochemical evidence of liver damage is consistent with intrahepatic induction of the receptor-mediated cell death genes *TRAIL* (*TNFSF10*), *Fas* (*FAS*) and *Fas ligand* (*FASLG*) and the cytotoxic effector gene *perforin* (*PRF1*) during high dose treatment (Table 3). These genes, as well as a death receptor signaling pathway (S8 Fig), were also significantly induced (on a group level) by low dose wIFN-α, consistent with liver enzyme elevations in some animals during this treatment period (Fig 4). Notably, although there was substantial induction of *TRAIL* expression (>17-fold) by high dose wIFN-α in two animals with a treatment response, one responder animal had only modest intrahepatic *TRAIL* induction (animal F1013; maximal 6-fold induction), and an animal with no treatment response had the greatest *TRAIL* induction (animal F1014; 118-fold) (S6 Table). This overall poor correlation of intrahepatic *TRAIL* with treatment response suggests that additional antiviral mechanisms may be required to control infection.

CD8^+^ T cells and NK cells have the potential to inhibit HBV infection by non-cytolytic mechanisms mediated by IFN-γ and TNF-α, as well as by killing infected cells via cytotoxic effector molecules. It is therefore notable that the two genes induced to the greatest degree in the liver by high dose wIFN-α treatment, *PLA2G2A* and *CXCL9*, are IFN-γ responsive genes [24] (Table 2). Furthermore, *PLA2G2A*, *CXCL9* and other IFN-γ inducible genes (as well as IFN-γ itself) are members of a subset of intrahepatic ISGs that correlated with wIFN-α dose (Figs 7A, Cluster 2, and S11). Strikingly, a large number of IFN-γ-regulated genes (e.g. MHC class I and II (*HLA*) genes, *CXCL9*) were also induced in the liver of chimpanzees during clearance of acute HBV infection [32]. In addition, although the on-treatment profile was not determined, MHC class I and II genes as well as *CXCL9* were also up-regulated prior to treatment in the liver of CHB patients that subsequently responded to pegylated IFN-α and adefovir treatment compared to non-responder patients [20]. Consistent with the association between blood IFN-γ expression and antiviral response (S5 Fig), high dose wIFN-α significantly induced intrahepatic *CXCL9* expression in animals with a treatment response, but not in those without an antiviral response (Fig 8C and S6 Table). These data indicate that IFN-γ-mediated, non-cytolytic mechanisms may play a role in the antiviral response to wIFN-α treatment. This is supported by the observation that the initial reduction in WHsAg and WHV DNA by high dose treatment in two responder animals (F1013 and F1022, weeks 7–15 and 7–11, respectively) occurred in the absence of substantial liver enzyme elevations (Fig 4). In both animals, there were subsequently modest increases in liver enzyme levels together with a further decrease in viral levels, suggesting that the antiviral response induced by high dose wIFN-α treatment is mediated by both cytolytic and non-cytolytic NK/T cell responses.

### Counter-regulatory mechanisms induced by IFN-α may limit treatment response

In addition to positive effects on antiviral immunity, wIFN-α also induced various counter-regulatory mechanisms that may have limited the antiviral response to treatment. Notably, intrahepatic mRNA levels of the inhibitory T cell receptor *PD-1* (*PDCD1*) and its ligand *PD-L1* (*CD274*) were significantly increased during wIFN-α treatment (Tables 2 and 3). Intrahepatic expression of *indoleamine 2*,*3-dioxygenase 1* (*IDO1*), which limits the availability of the essential amino acid tryptophan and produces immunosuppressive kynurenine to locally suppress T cells [33], was also significantly increased by wIFN-α treatment (Table 3). Furthermore, high dose wIFN-α modestly elevated intrahepatic *FOXP3* mRNA levels (Table 2), which suggests treatment-associated migration and/or proliferation of T regulatory cells (Tregs) that may negatively regulate CD8^+^ T cell and NK cell function. In contrast, expression of *IL-10* (*IL10*) and *TGF-β* (*TGFB1*), immunosuppressive cytokines produced by Tregs and various other cells, was not significantly modulated by treatment (Table 3).

## Discussion

Recombinant IFN-α has been used to treat CHB for over 20 years, but the molecular basis of treatment response remains poorly understood [3]. Previous transcriptome analyses have shown there are important parallels between the immune response to WHV in woodchucks and HBV in man [24], and that self-limiting hepadnavirus infection in woodchucks and chimpanzees share key immunological features [25]. Together these studies suggest that the woodchuck is a relevant model to study the mechanisms that govern antiviral response to IFN-α. Consequently, we characterized the intrahepatic transcriptional profile of WHV chronic carrier woodchucks during treatment with recombinant woodchuck IFN-α. Treatment with wIFN-α produced variable antiviral effects, inducing multi-log reduction in serum WHV DNA and WHsAg in a subset of animals, and sustained WHsAg loss and seroconversion to anti-WHsAb in one animal, while not exerting antiviral effects in other animals. Importantly, the variability and degree of antiviral response in these animals are comparable to those observed with CHB patients treated with pegylated IFN-α [2,34]. Furthermore, viral rebound in the WHV-infected woodchucks was typically observed following cessation of wIFN-α treatment, consistent with the low rate of durable HBsAg loss in patients treated with IFN-α [2]. Together, these data reveal important parallels between the IFN-α treatment response of chronic hepadnavirus infection in woodchucks and man, establishing the translational value of the woodchuck model for characterizing the immune correlates of IFN-α treatment response.

Since various studies have demonstrated that IFN-α can directly inhibit HBV [6,11,13], a striking finding of this study was that the antiviral response to wIFN-α did not correlate with the intrahepatic induction of the majority of antiviral ISGs. Since WHV is sensitive to the direct antiviral effects of wIFN-α in vitro [23,35], our data suggest that IFN-induced antiviral effectors of WHV do not play a key role in the antiviral response to treatment in vivo. However, there are several important caveats to consider. Firstly, since there are a large number of antiviral ISGs, and not all were available in the woodchuck transcriptome (e.g. APOBEC3A), intrahepatic expression of antiviral ISGs that were not evaluated in this study may correlate with treatment response. In addition, since intrahepatic transcriptional analysis was restricted to 6 hours post-dose, it is possible that the expression of certain ISGs with slower induction kinetics may be associated with the antiviral response to IFN-α. Secondly, although low dose wIFN-α induced intrahepatic ISG expression but not a significant antiviral response, it is conceivable that prolonged ISG expression (7 weeks) by low dose treatment played an important role in the antiviral response subsequently induced by higher dose wIFN-α. Finally, transcriptional analysis of whole biopsy tissue cannot define cell-specific ISG expression, which may be important in treatment response [36]. This is also an important caveat if hepatocytes and non-parenchymal cells (e.g. Kupffer cells) display markedly different sensitivity to ISG induction, since it may preclude accurate correlation of treatment response with induction of antiviral ISGs in infected cells using whole biopsy tissue.

The significant difference in ISG induction by low and high dose wIFN-α in the blood but not the liver of woodchucks chronically infected with WHV is noteworthy considering a recent study demonstrating that HBV can inhibit IFN-α signaling in human hepatocytes [37]. This suggests that WHV may limit (although not abrogate) wIFN-α signaling in woodchuck hepatocytes. Alternatively, induction of *USP18*, *SOCS1* and *SOCS3* (Table 3) and/or other inhibitors of IFN-α/β receptor signaling may limit the intrahepatic ISG response to wIFN-α treatment. Since liver biopsies were not taken after wIFN-α treatment of WHV-negative animals, and there is currently no sensitive, quantitative wIFN-α ELISA (see Methods), additional studies will be required to determine whether there are significant differences in PK-PD responses to wIFN-α treatment in WHV-negative and WHV-infected animals.

In contrast to intrahepatic ISG expression, the expression of other gene sets showed a correlation with antiviral response. Both NK/T cell and IFN-γ transcriptional signatures in the liver were increased in animals with antiviral response to wIFN-α treatment. The peak antiviral response was also associated with liver enzyme elevations in some (although not all) animals. Collectively these data suggest that the antiviral response induced by wIFN-α treatment was mediated by both cytolytic and non-cytolytic NK/T cell responses. The correlation of liver injury biomarkers with antiviral response is notable because host-induced ALT flares are associated with IFN-α treatment response in CHB patients [38]. The association of intrahepatic NK cell and IFN-γ transcriptional signatures with antiviral response to treatment is also striking because NK cells in CHB patients have a markedly impaired capacity to produce IFN-γ [39,40]. This dysfunctional phenotype can be reversed (at least in NK cells in the periphery) by treatment with IFN-α [17], which suggests that NK cell IFN-γ production may represent a common mechanism of IFN-α antiviral response to chronic hepadnavirus infection in woodchucks and man. Clearance of acute HBV infection in chimpanzees is also characterized by an intrahepatic IFN-γ transcriptional signature [32], suggesting that there are important parallels between the immunological mechanisms of natural clearance of HBV and those induced by IFN-α treatment.

Recent studies have revealed that IFN-α treatment does not improve peripheral HBV-specific CD8^+^ T cell responses [16–18]. In view of the aforementioned NK cell activation by IFN-α, this failure to augment virus-specific CD8^+^ T cell responses may be explained, at least in part, by the observation that NK cells can directly kill HBV-specific CD8^+^ T cells via TRAIL and other mechanisms [41]. The induction of an intrahepatic NK signature as well as *TRAIL* expression suggests that WHV-specific CD8^+^ T cell responses may be inhibited by similar mechanisms during wIFN-α treatment. Conversely, IFN-induced protection of antiviral CD8^+^ T cells might limit NK regulation of T cell immunity in this setting [42,43], consistent with the induction of an intrahepatic T cell transcriptional signature coupled with significant elevation of *T-bet* (*TBX21*) mRNA during wIFN-α treatment. In addition to potentially inducing NK cell killing of virus-specific CD8^+^ T cells, wIFN-α treatment induced various counter-regulatory mechanisms, including intrahepatic *PD-1* (*PDCD1*) and *PD-L1* (*CD274*) expression, which may also have limited antiviral CD8^+^ T cell function in the liver. However, it is important to the note that a limitation of the woodchuck model is that it is challenging to confirm that changes in gene expression are associated with corresponding changes in protein levels and/or cellular function. This is particularly important for characterization of CD8^+^ T cell specificity in the context of wIFN-α treatment, since studies in HBV transgenic mice as well as CHB patients indicate that antigen-nonspecific inflammatory cells (including nonvirus-specific CD8^+^ T cells) can accumulate to high levels in the liver under inflammatory conditions [44,45]. Unfortunately, blood volume and biopsy material limitations precluded functional analysis of WHV-specific CD8^+^ T cells in the current study. In addition, the lack of woodchuck-specific immunological reagents prevented immunophenotyping of WHV-specific CD8^+^ T cells by flow cytometry. Attempts to develop high-quality monoclonal antibodies against woodchuck CD56 and CD8a to enable detection of NK and CD8^+^ T cells, respectively, by immunohistochemistry were also not successful. Therefore, additional studies in immunocompetent models of natural infection and/or CHB patient biopsies will be required in order to define the relative contribution of intrahepatic NK and virus-specific CD8^+^ T cells to IFN-α treatment response.

In summary, by studying recombinant IFN-α in an immunocompetent animal model of CHB, this study provided new insights into the immune mechanisms that mediate the antiviral response to treatment. In addition, various immune pathways were identified that may act to limit treatment response. These findings have important implications for the design of new therapeutics for CHB, and also provide rationale for evaluating combinations of immunotherapeutic agents currently in development.

## Materials and Methods

### Expression, purification and analytical characterization of woodchuck interferon

The sequence of woodchuck IFN-α5 (wIFN-α) has previously been described [26]. Recombinant wIFN-α was expressed by transient transfection of human embryonic kidney (HEK) 293F cells using the FreeStyleTM 293 expression system according to the manufacturer’s instructions (Invitrogen, Inc., Carlsbad, CA). Culture supernatant was filtered and then purified by two chromatographic steps. Firstly, after adjusting to pH 6.0 with 50 mM KH_2_PO_4_, pH 5.0, the sample was loaded on a 5 mL SP HP Hi Trap (GE Healthcare, Little Chalfont, Buckinghamshire, UK) that had been pre-equilibrated with 50 mM KH_2_PO_4_, pH 6.0. The wIFN-α was then eluted with a 17 column-volume salt gradient from 0–500 mM NaCl. Fractions were analyzed via SDS-PAGE and wIFN-containing fractions were pooled. Secondly, size exclusion chromatography on Superdex 75 (GE Healthcare, Little Chalfont, Buckinghamshire, UK) was performed in 20 mM His/HCl, 140 mM NaCl pH 6.0. The eluted wIFN-α was filtrated with a 0.22 μM syringe filter and stored at -80°C. The wIFN-α concentration was determined by measuring optical density (OD) at 280 nm. Purity and monomer content were confirmed by SDS-PAGE and SE-HPLC, respectively, and the integrity of the wIFN-α amino acid backbone was verified by Nano Electrospray QTOF mass spectrometry. The protein was kept in a storage buffer (20 mM His/HCl, 140 mM NaCl pH 6.0) prior to dosing. The endotoxin level of the wIFN-α preparation was <0.454 EU/mL. The in vitro biological activity of wIFN-α was confirmed by dose-dependent induction of mRNA levels of the interferon-stimulated genes (ISGs) *Mx1* and *OAS1* in woodchuck PBMCs (n = 2 animals) treated with 0.1, 1 and 10 μg/mL wIFN-α.

### Ethics statement

The animal protocol and all procedures involving woodchucks were approved by the Georgetown University IACUC (Protocol Number: 11–006) and adhered to the national guidelines of the Animal Welfare Act, the Guide for the Care and Use of Laboratory Animals, and the American Veterinary Medical Association.

### Single dose wIFN-α study in WHV-negative woodchucks

All woodchucks used in this study were obtained from Northeastern Wildlife. Prior to the study, male woodchucks were confirmed negative for WHV surface antigen (WHsAg) and for antibodies against WHsAg (anti-WHsAb) and WHV core antigen (anti-WHc). Animals were assigned to four groups (n = 3/group) using stratification based on body weight, clinical biochemistry and hematology. Animals received a single subcutaneous dose of 2, 20 or 200 μg wIFN-α, or a placebo control (all n = 3/group). Various measurements (body weight, body temperature, clinical serum chemistries, and CBCs) were obtained to monitor drug safety.

### Repeat dose wIFN-α study in WHV carrier woodchucks

All woodchucks used in this study were obtained from Northeastern Wildlife. These woodchucks were born in captivity and were infected at 3 days of age with the cWHV7P2a inoculum containing WHV strain WHV7-11. cWHV7P2a has the same biological and virological characteristics as the cWHV7P2 inoculum as both were derived from cWHV7P1 [46]. Chronically infected animals were all anti-WHs negative, with detectable serum WHV DNA, WHsAg and anti-WHc at approximately 1 year post-infection. Absence of liver tumors in woodchucks with low GGT was confirmed by ultrasonography. Chronic WHV carrier woodchucks were assigned and stratified by gender, body weight, and by pretreatment serum markers (WHsAg and WHV DNA concentrations, serum GGT and SDH activities) into treatment and placebo groups (n = 12/group). The study design and sampling scheme are summarized in Fig 1.

### Pharmacokinetics (PK) of wIFN-α

The PK of wIFN-α was not measured due to the lack of a suitable analytical method. Although a wIFN-α ELISA has previously been described [27], it was discovered during method development that one of the antibodies likely recognized the 6xHis tag of the antigen used for immunization, which was not present in our preparation of wIFN-α. Despite extensive screening of available anti-human, anti-macaque, anti-mouse and anti-pig IFN-α antibodies (PBL, Piscataway, NJ), as well as additional anti-woodchuck IFN-α antibodies (Digna Biotech, Pamplona, Spain), none were identified that robustly detected wIFN-α in an ELISA format.

### WHV parameters

Serum WHV DNA was quantified by two different methods depending on concentration: dot blot hybridization or real time PCR assay on a 7500 Real Time PCR System instrument (Applied Biosystems, Foster City, CA) as described previously [47]. Serum WHsAg and anti-WHsAb were measured by WHV-specific enzyme immunoassays as described [48]. Liver WHV RNA was measured quantitatively by Northern blot hybridization as previously described [49]. Liver WHV DNA replicative intermediates (RI) and WHV cccDNA were quantitatively determined by Southern blot as previously described [50].

### Woodchuck transcriptome assembly

The revised woodchuck transcriptome assembly (version 2) consists of a previous assembly (version 1), generated with Roche-454 sequencing data [24], that was merged with newly assembled contiguous transcripts (contigs) from Illumina sequencing data of the 24 animals from the current study (n = 12 placebo, n = 12 wIFN-α treated). The main improvement of version 2 over version 1 is that the sequencing depths of the Illumina data is significantly higher than that of 454 and therefore resulted in a higher dynamic range and increased number of genes as compared to assembly version 1 (S2 Table). The assembly method of transcriptome version 2 consisted of three stages: 1) initial contig assembly, 2) contig annotation and 3) contig refinement. First, Illumina RNA-Seq paired-end reads from liver samples were assembled using Trinity [51] (release 2011-08-20). The obtained contigs were further refined and merged by applying the sequence assembly algorithm PHRAP [52]. As a result, the number of contigs was reduced by about 25% and the contig lengths were increased. Second, all contigs were subjected to an in-house developed gene annotation pipeline which performs sequence homology searches within reference transcript databases from other species. First, woodchuck contigs were mapped to transcripts from RefSeq reference database containing human, mouse, and rat transcripts using BLAST [53], with a 1.e-5 E-value cutoff. Matches with the highest BLAST scores were further pair-wise aligned by applying the Needleman-Wunsch algorithm [54] in order to obtain more accurate alignments and to calculate the sequence identities (i.e. number of identical nucleotides in percentage of alignment length) between RefSeq transcripts and woodchuck contigs. If the identity difference between the two best hits exceeded 25%, then the top gene was used for contig annotation. Only contigs that could be mapped to known mouse, rat or human genes were used for further data processing. Because the assembly often contained more than one contig per gene, a final sequence refinement was then performed to remove redundancies. Contigs annotated with identical genes were subjected to the CAP3 assembler [55], and as a result, the number of contigs was further reduced and the sequence lengths of numerous contigs were increased.

### RNA-Seq analysis

Sequencing libraries were created using Illumina’s TruSeq RNA sample preparation kit (San Diego, CA) according to manufacturer’s protocol. Total RNA was purified using oligo(dT) magnetic beads, fragmented, and reverse-transcribed using SuperScript II (Invitrogen, Inc., Carlsbad, CA) to synthesize first strand cDNA. After second strand synthesis, Illumina specific adapters containing unique barcodes were ligated to the ends of the double-stranded cDNA. Fragments containing adapters on both ends were then enriched and amplified with PCR, quantified with qPCR, and run on the Agilent Bioanalyzer DNA-1000 chip to estimate fragment size. Samples were then multiplexed and sequenced on the Illumina 2500. The data was demultiplexed using CASAVA and run through FastQC (http://www.bioinformatics.babraham.ac.uk/projects/fastqc/) to assess sequencing data quality. Paired-end 50 nucleotide read data from mRNA-Seq were mapped against the revised woodchuck transcriptome with Bowtie2 [56] and prioritized for concordant paired alignments with unique hits. The resulting SAM/BAM files were processed with SAMtools [57] to yield count data that was normalized and processed by DESeq [58] for differential expression analysis and subsequent pattern recognition and pathway analysis. Multiple testing correction was performed using the method of Benjamini and Hochberg [59]. Principal component analysis was performed with Partek Genomics version 6.6beta (Partek, St. Louis, MO). Heatmaps of the expression data were generated by unsupervised hierarchical clustering of least square means expression values, after z-score normalization across samples. The enrichment of differential genes relative to the gene modules described previously [29] was calculated with R version 2.13.2 (http://www.r-project.org) using the humanized gene symbols for the woodchuck genes. Gene Set Enrichment Analysis (GSEA) was performed as previously described [60], with ranks determined by the multiplicative product of the fold-change and–log(FDR) values for each gene. Weighted gene coexpression network analysis (WGCNA) [61] was performed within the R statistics environment. Pathway analysis was performed using Ingenuity Pathway Analysis (Ingenuity Systems, Redwood City, CA).

### Quantitative RT-PCR

Total RNA was isolated using the RNeasy Mini Kit (Qiagen Inc., Redwood City, CA) with on-column DNase digestion using the RNase-Free DNase Set (Qiagen Inc., Redwood City, CA). Following reverse transcription into cDNA with the Transcriptor First Strand cDNA Synthesis Kit (Roche Applied Sciences, Indianapolis, IN), samples were analyzed by real time PCR on a 7500 Real Time PCR System instrument (Applied Biosystems, Inc., Foster City, CA) using EagleTaq Universal Master Mix (Roche Applied Sciences, Indianapolis, IN). Target gene expression was normalized to 18S rRNA expression. The primers and probes used in this study are displayed in S7 Table, or have previously been described [24,62–65]. Note that, although there was insufficient mRNA from biopsy samples to perform extensive qRT-PCR validation of gene expression, in contrast to microarray, RNA-Seq has high concordance with qRT-PCR data [28].

## Supporting Information

S1 Fig Dose-dependent pharmacodynamic response to wIFN-α in healthy adult male woodchucks. qRT-PCR data expressed as fold-change relative to pre-dose. Circles indicate the mean of each dose group (n = 3 animals), and the errors bars represent the standard error of the mean.
(TIF)

S2 Fig Serum (a) WHsAg and (b) WHV DNA for individual wIFN-α and placebo group animals that survived until end-of-study. Top panel: placebo group (n = 9); bottom panel: wIFN-α group (n = 7). Note that seven animals (three in the placebo group, four in the wIFN-α group) died during the study and one animal in the wIFN-α group (M1004) was excluded from the analysis since it was likely naturally clearing WHV as the study began (see Table 1). Change from baseline was calculated relative to week -3 (pre-treatment baseline). Line color denotes response to wIFN-α treatment: red indicates responder (R, ≥1 log_10_ reduction in WHsAg at week 15 and week 23); green indicates partial responder (PR, ≥1 log_10_ reduction in WHsAg at week 15 but not week 23); blue indicates non-responder (NR, <1 log_10_ reduction in WHsAg at weeks 15 and 23). Note: the WHsAg level for animal M1002 was ≤ LLOD (20 ng/mL) at week 16, and for animal F1022 at weeks 12–23; the LLOD was used to estimate the WHsAg decline for these animals at these timepoints.
(TIF)

S3 Fig Intrahepatic (a) WHV cccDNA, (b) WHV replicative intermediate (RI) DNA and (c) WHV RNA for wIFN-α and placebo animals. Left panels: circles indicate the mean of each group, and the errors bars represent the standard error of the mean. Data from all animals was included in the plots, with the exception of one animal (M1004) in the wIFN-α group (see Table 1). Right panels: plots of individual animals treated with wIFN-α that survived until end-of-study (n = 7). Line colors denote response to treatment (as measured by WHsAg) and are described in S2 Fig. Change from baseline was calculated relative to week -3 (pre-treatment baseline). Note that the data from animal M1002 at week 25 (end-of-study for this responder animal) was plotted instead at week 23 (end-of-study for most animals) for ease of data comparison.
(TIF)

S4 Fig Serum WHsAg and liver histology score for individual wIFN-treated animals. The liver histology score was derived from the lobular sinusoidal hepatitis score combined with the mean of the portal hepatitis score (n = 1–5 portal tracts examined). A composite histology score of >0–2 indicates mild hepatitis, >2–4 indicates moderate hepatitis and >4 indicates marked to severe hepatitis. The treatment response group classifications (a-d) are described in Table 1. *Animals died prior to end-of-study. ND: not determined.
(TIF)

S5 Fig Whole blood gene expression in relation to treatment response. qRT-PCR data expressed as fold-change relative to week 0 pre-dose (pre-treatment baseline). The bar height indicates the mean maximal fold-change during weeks 0–15 (at 6 hours post-dose) for the placebo (P) group and each wIFN-α treatment response group, and the error bars represent the standard error of the mean. The symbols immediately above the bars denote the level of statistical significance relative to the placebo group: *p<0.05; **p<0.01; ***p<0.001. The p-values above the horizontal lines indicate the level of statistical significance between the various response groups (defined in Table 1). Statistical significance was calculated with log-transformed values by one-way ANOVA with Tukey's multiple comparison correction.
(TIF)

S6 Fig wIFN-α treatment transiently induced changes in the liver transcriptome of animals chronically infected with WHV. Principal component (PC) analysis of normalized liver gene expression data for animals at pre-treatment (baseline; week-3), during treatment (weeks 0–15, all 6 hours post-dose) and post-treatment (follow-up; weeks 17–25). Note that only a subset of animals were sampled at week 17 (n = 2, both placebo) and week 25 (n = 2, both wIFN-α) due to premature termination and extended follow-up, respectively. The two sets of samples that were substantially differentiated by this analysis (i.e. separated by first component, PC#1) are highlighted by the red and green ellipses, and are described by the text positioned above.
(PDF)

S7 Fig Schematic description of the strategy for the identification of genes associated with response to wIFN-α treatment. Pairwise comparisons of low dose (20 μg; weeks 0 and 3) and high dose (100 μg; week 15) wIFN-α group relative to time-matched placebo controls. The dashed boxes at the top of the figure provide details of the samples (treatment group, wIFN-α dose, study week, number of animals) included in the DEG selection. The week 15 timepoint was selected for high dose wIFN-α due to its close proximity to the serum WHV DNA and WHsAg nadir (week 16). The name of each gene set is displayed below the Venn diagram. ^†^DEGs that were significantly modulated in either W0^IFN^ vs. W0^PBO^ or W3^IFN^ vs. W3^PBO^ were also excluded (n = 286 total). The transcriptional signatures for "Low & High Dose" (n = 775) and "High Dose" (n = 468) are described in Figs 8 and S8, respectively. No gene signatures were significantly enriched for "Low Dose" (n = 29). PBO: placebo, W: week, DEG: differentially expressed gene, FC: fold-change.
(PDF)

S8 Fig Intrahepatic transcriptional signatures associated with both low dose and high dose wIFN-α treatment. Analysis of genes (n = 775) induced by both low dose (20 μg) and high dose (100 μg) wIFN-α (S7 Fig, “Low & High Dose”). Top panel: modular analysis of intrahepatic gene expression, as described in Fig 6. Only modules with enrichment greater than 10% at one or more time-point are displayed. Bottom panel: top canonical pathways identified by Ingenuity Pathway Analysis. Pathway enrichment was calculated with the Fisher’s exact test with multiple testing correction by the Benjamini and Hochberg method. The–log(*p*-value) for *p* = 0.05 and *p* = 0.01 significance levels are indicated.
(PDF)

S9 Fig Characterization of intrahepatic transcriptional signature associated with response to wIFN-α treatment. Top canonical pathways identified by Ingenuity Pathway Analysis for (a) WGCNA module 1 (n = 187 genes), and (b) WGCNA module 2 (n = 379 genes) (see S5 Table) from baseline to week 15 for the treatment group relative to the placebo group. Pathway enrichment was calculated with the Fisher’s exact test with multiple testing correction by the Benjamini and Hochberg method. The–log(p-value) for p = 0.05 and p = 0.01 significance levels are indicated.
(TIF)

S10 Fig Modular analysis of intrahepatic transcriptional signatures in individual animals receiving wIFN-α treatment. Animal numbers by response group: NR: M1012 (on left at each timepoint) and F1014 (on right at each timepoint); PR: M1003 (left) and F1018 (right); R: M1002 (left), F1013 (middle) and F1022 (right). Note that there was no week 19 sample for the responder group animal M1002. Spot intensity (red: over-expressed; blue: under-expressed) denotes the percentage of transcripts significantly changed in each module (M), as described in Fig 6. The functional interpretation of each module is displayed on the right. The percentage of changed transcripts was determined by the GSEA enrichment score (ES) between week 15 and baseline for each sample. Enrichment scores for gene modules passing the GSEA FDR threshold <0.05 were scaled for plotting. Only modules for which the leading edge genes were enriched (>10% of module genes, p<0.05 by Fisher’s exact test) are displayed. The horizontal bars together with the week (W) numerators indicate the study stage, as described in Fig 1.
(PDF)

S11 Fig Intrahepatic expression of a subset of ISGs correlates with wIFN-α dose and treatment response. Unsupervised hierarchical clustering of genes from cluster 2 of Fig 7A. The colors immediately above the heatmap indicate animals that were non-responders (NR, n = 2), partial responders (PR, n = 2) or responders (R, n = 3) to wIFN-α treatment. Note that there was no week 19 sample for the responder group animal M1002 and the week 25 sample (end-of-study for this responder animal) was included at week 23 (end-of-study for most animals) for ease of data comparison. Heatmap columns represent samples from individual animals collected at the indicated times, and rows represent different genes (n = 35). Red and blue coloring of cells represents high and low expression levels (normalized count data), respectively, as indicated by the scale bar for log_2_ normalized values.
(PDF)

S1 Table Histologic and antibody response to wIFN-α and placebo treatment. The liver histology score was derived from the lobular sinusoidal hepatitis score combined with the mean of the portal hepatitis score (n = 1–5 portal tracts examined). A composite histology score of >0–2 indicates mild hepatitis, >2–4 indicates moderate hepatitis and >4 indicates marked to severe hepatitis. Dashed line indicates no sample at week -3 (baseline). The LLOD for the anti-WHs assay was 100 StdU/mL. Values between 100–200 StdU/mL were considered trace; 200–300 very low; 300–500 low; 500–2,000 moderate; and greater than 2,000 were considered high (i.e. indicates potential seroconversion). Animals M1004 and F1022 had anti-WHs values >500 StdU/mL at weeks 5–25 and 13–23, respectively. The response group classifications are described in Table 1.
(DOCX)

S2 Table Comparison of the original (version 1) and revised (version 2) woodchuck transcriptome assemblies. Contigs were mapped with BLAST and E-value cut-off of 1.e-10 to human, mouse and rat coding and non-coding RefSeq transcripts. The numbers in parentheses in the first column are the total number of genes or transcripts in the corresponding RefSeq databases as of November 2014. The version 1 transcriptome assembly is described in reference [24]. The version 2 transcriptome assembly is described in the Methods.
(DOCX)

S3 Table Sources of annotated ISGs for characterization of the intrahepatic IFN response in woodchucks treated with wIFN-α. A total of 233 ISGs were compiled from these gene sets, of which 209 were present in version 2 of the woodchuck transcriptome. The intrahepatic expression of these ISGs in woodchucks treated with wIFN-α is displayed in Fig 7A.
(DOCX)

S4 Table qRT-PCR quantitation of select intrahepatic ISGs. qRT-PCR data expressed as fold-change relative to week -3 (pre-treatment baseline). Week 0: sample collected 6 hours post-first dose of 20 μg wIFN-α or placebo. Week 7: sample collected 6 hours post-first dose of 100 μg wIFN-α or 23^rd^ dose of placebo. ND: not determined (animal died prior to biopsy time-point (see Table 1), insufficient sample available or mRNA quality not appropriate for analysis). Intrahepatic expression of these genes at week 0 and week 7 was not significantly different (Fig 7B). The response group classifications are described in Table 1.
(DOCX)

S5 Table Correlation of different gene sets with WHsAg and WHV DNA. Gene sets (modules) were identified by unsupervised WGCNA analysis and are sorted by inverse correlation with WHsAg, i.e. module 1 has highest negative r value. Cell shaded grey if correlation (negative or positive) was statistically significant (*p*<0.05). WGCNA modules with >1000 genes were not included since the size precluded accurate determination of key gene signatures.
(DOCX)

S6 Table qRT-PCR quantitation of select intrahepatic genes. qRT-PCR data expressed as fold-change relative to week -3 (pre-treatment baseline). Week 0: sample collected 6 hours post-first dose of 20 μg wIFN-α or placebo. Week 7: sample collected 6 hours post-first dose of 100 μg wIFN-α or 23^rd^ dose of placebo. Week 15: sample collected 6 hours post-last dose of 100 μg wIFN-α or 45^th^ dose of placebo. ND: not determined (animal died prior to biopsy time-point (see Table 1), insufficient sample available or mRNA quality not appropriate for analysis). The response group classifications are described in Table 1.
(DOCX)

S7 Table Oligonucleotides used for qRT-PCR. F: forward primer; R: reverse primer; P: probe. Note the Hugo gene symbol for TRAIL is TNFSF10.
(DOCX)

S8 Table List of genes in each of the gene sets described in S7 Fig. FC: fold-change. (a) Low dose only (n = 29). (b) Low and high dose (n = 775). (c) High dose only (n = 468).
(XLSX)
