# Correction: Intrahepatic Transcriptional Signature Associated with Response to Interferon-α Treatment in the Woodchuck Model of Chronic Hepatitis B

**DOI:** 10.1371/journal.ppat.1005541

**Published:** 2016-03-29

**Authors:** Simon P. Fletcher, Daniel J. Chin, Lore Gruenbaum, Hans Bitter, Erik Rasmussen, Palanikumar Ravindran, David C. Swinney, Fabian Birzele, Roland Schmucki, Stefan H. Lorenz, Erhard Kopetzki, Jade Carter, Miriam Triyatni, Linta M. Thampi, Junming Yang, Dalal AlDeghaither, Marta G. Murreddu, Paul Cote, Stephan Menne

The seventeenth author's name is spelled incorrectly. The correct name is: Marta G. Murreddu.
