# Supplementary figures and images for: Intrahepatic Transcriptional Signature Associated with Response to Interferon-α Treatment in the Woodchuck Model of Chronic Hepatitis B
Source: PLoS Pathog. 2015 Sep 9;11(9):e1005103. doi: 10.1371/journal.ppat.1005103 (PMC4564242; doi:10.1371/journal.ppat.1005103)

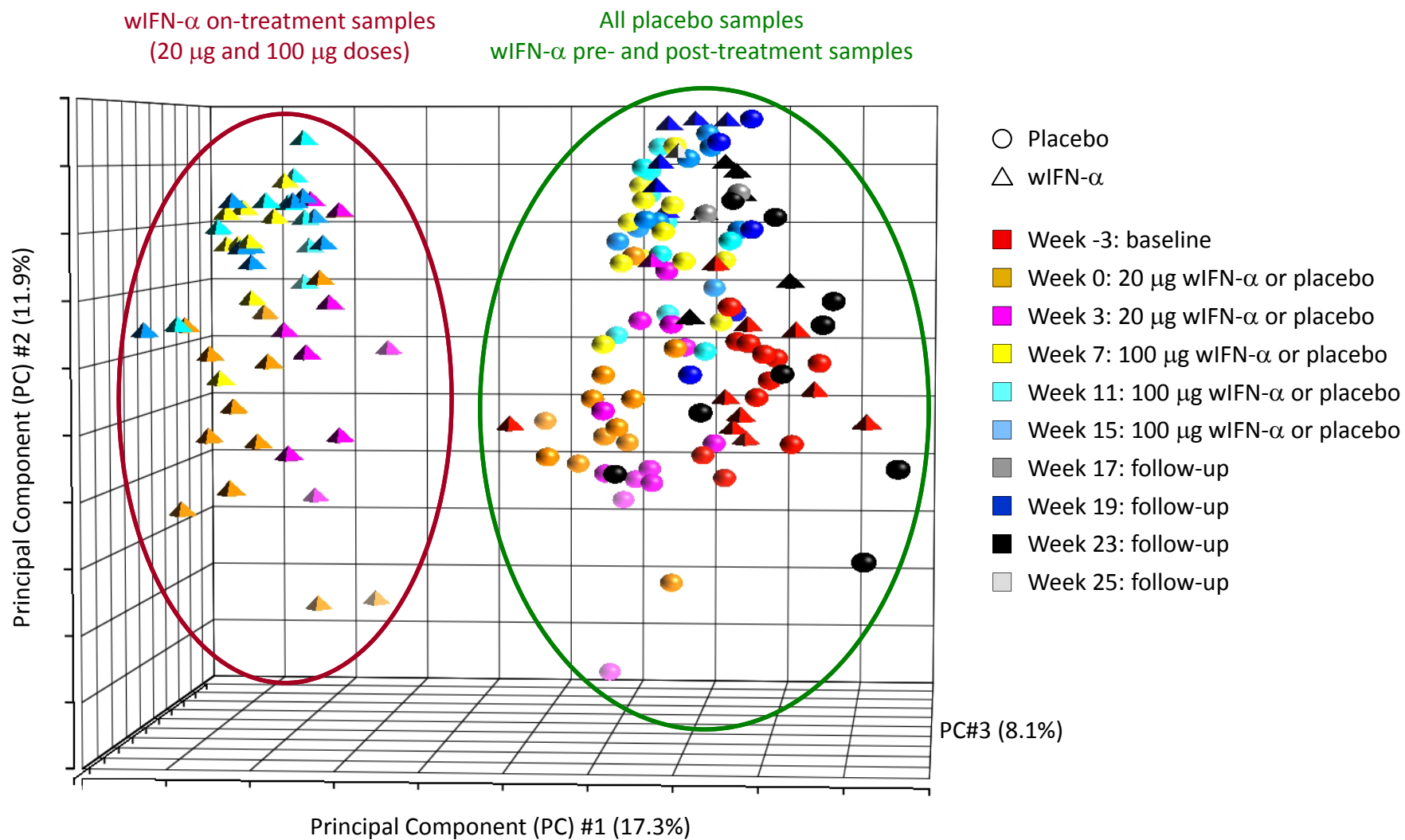

Supplement: S6 Fig — Principal component (PC) analysis of normalized liver gene expression data for animals at pre-treatment (baseline; week-3), during treatment (weeks 0–15, all 6 hours post-dose) and post-treatment (follow-up; weeks 17–25). Note that only a subset of animals were sampled at week 17 (n = 2, both placebo) and week 25 (n = 2, both wIFN-α) due to premature termination and extended follow-up, respectively. The two sets of samples that were substantially differentiated by this analysis (i.e. separated by first component, PC#1) are highlighted by the red and green ellipses, and are described by the text positioned above. (PDF) [file ppat.1005103.s006.pdf]

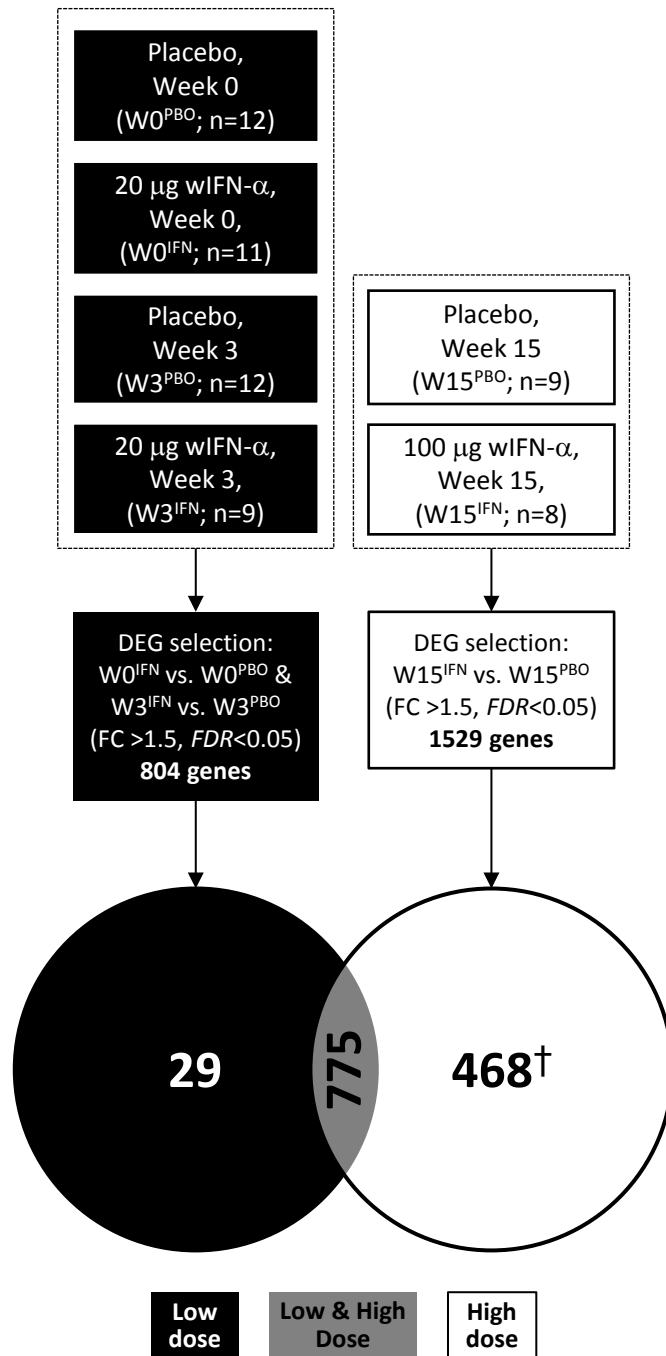

Supplement: S7 Fig — Pairwise comparisons of low dose (20 μg; weeks 0 and 3) and high dose (100 μg; week 15) wIFN-α group relative to time-matched placebo controls. The dashed boxes at the top of the figure provide details of the samples (treatment group, wIFN-α dose, study week, number of animals) included in the DEG selection. The week 15 timepoint was selected for high dose wIFN-α due to its close proximity to the serum WHV DNA and WHsAg nadir (week 16). The name of each gene set is displayed below the Venn diagram. †DEGs that were significantly modulated in either W0IFN vs. W0PBO or W3IFN vs. W3PBO were also excluded (n = 286 total). The transcriptional signatures for "Low & High Dose" (n = 775) and "High Dose" (n = 468) are described in Figs 8 and S8, respectively. No gene signatures were significantly enriched for "Low Dose" (n = 29). PBO: placebo, W: week, DEG: differentially expressed gene, FC: fold-change. (PDF) [file ppat.1005103.s007.pdf]

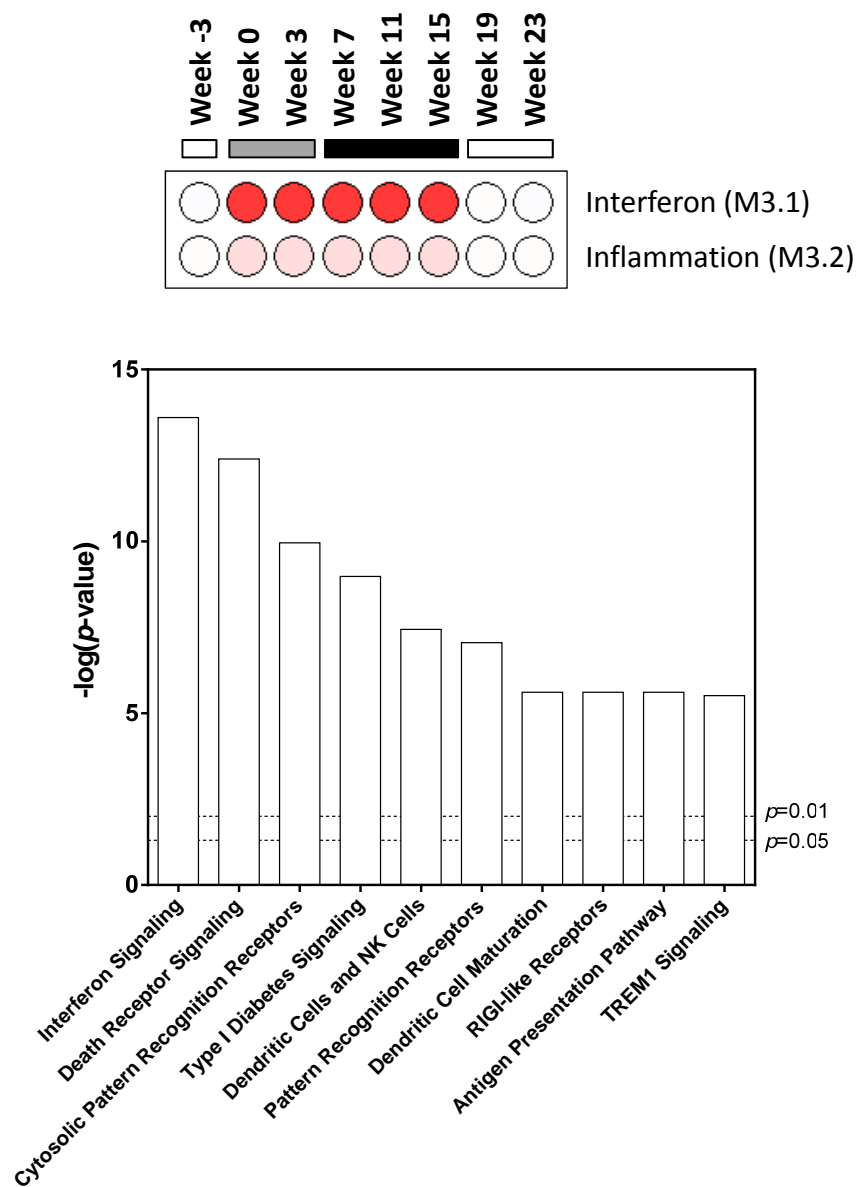

Supplement: S8 Fig — Analysis of genes (n = 775) induced by both low dose (20 μg) and high dose (100 μg) wIFN-α (S7 Fig, “Low & High Dose”). Top panel: modular analysis of intrahepatic gene expression, as described in Fig 6. Only modules with enrichment greater than 10% at one or more time-point are displayed. Bottom panel: top canonical pathways identified by Ingenuity Pathway Analysis. Pathway enrichment was calculated with the Fisher’s exact test with multiple testing correction by the Benjamini and Hochberg method. The–log(p-value) for p = 0.05 and p = 0.01 significance levels are indicated. (PDF) [file ppat.1005103.s008.pdf]

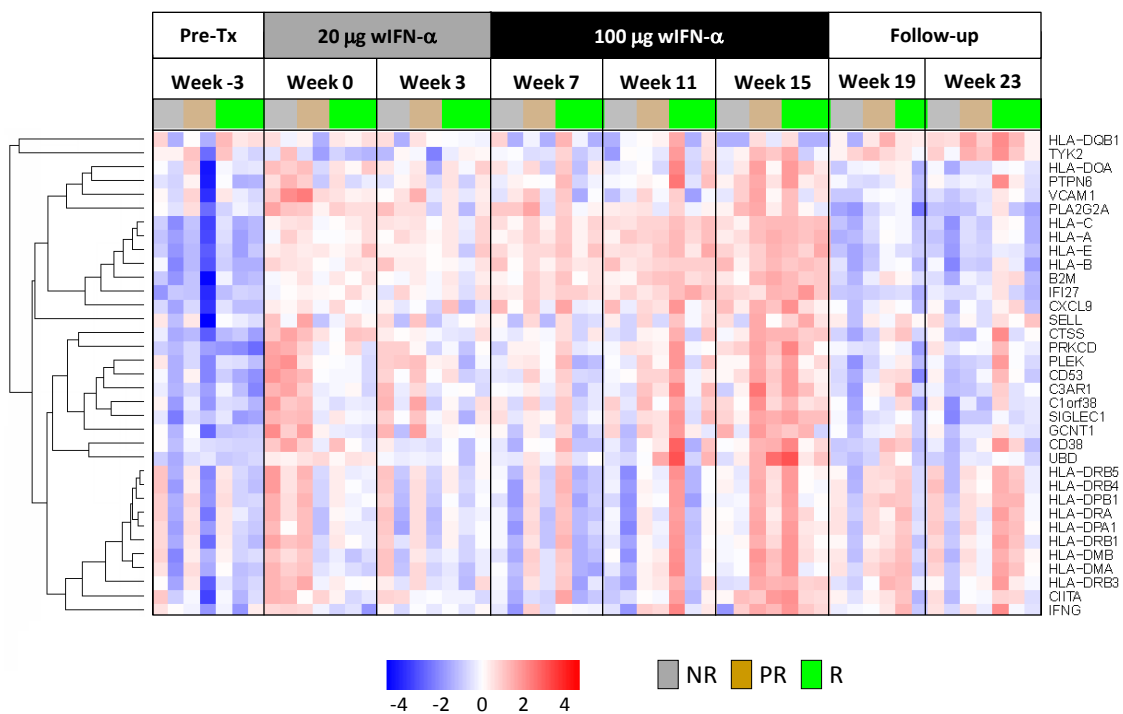

Supplement: S11 Fig — Unsupervised hierarchical clustering of genes from cluster 2 of Fig 7A. The colors immediately above the heatmap indicate animals that were non-responders (NR, n = 2), partial responders (PR, n = 2) or responders (R, n = 3) to wIFN-α treatment. Note that there was no week 19 sample for the responder group animal M1002 and the week 25 sample (end-of-study for this responder animal) was included at week 23 (end-of-study for most animals) for ease of data comparison. Heatmap columns represent samples from individual animals collected at the indicated times, and rows represent different genes (n = 35). Red and blue coloring of cells represents high and low expression levels (normalized count data), respectively, as indicated by the scale bar for log2 normalized values. (PDF) [file ppat.1005103.s011.pdf]
