# Supplementary material for: Intrahepatic Transcriptional Signature Associated with Response to Interferon-α Treatment in the Woodchuck Model of Chronic Hepatitis B
Source: PLoS Pathog. 2015 Sep 9;11(9):e1005103. doi: 10.1371/journal.ppat.1005103 (PMC4564242; doi:10.1371/journal.ppat.1005103)
Supplement: S10 Fig — Animal numbers by response group: NR: M1012 (on left at each timepoint) and F1014 (on right at each timepoint); PR: M1003 (left) and F1018 (right); R: M1002 (left), F1013 (middle) and F1022 (right). Note that there was no week 19 sample for the responder group animal M1002. Spot intensity (red: over-expressed; blue: under-expressed) denotes the percentage of transcripts significantly changed in each module (M), as described in Fig 6. The functional interpretation of each module is displayed on the right. The percentage of changed transcripts was determined by the GSEA enrichment score (ES) between week 15 and baseline for each sample. Enrichment scores for gene modules passing the GSEA FDR threshold <0.05 were scaled for plotting. Only modules for which the leading edge genes were enriched (>10% of module genes, p<0.05 by Fisher’s exact test) are displayed. The horizontal bars together with the week (W) numerators indicate the study stage, as described in Fig 1. (PDF) [file ppat.1005103.s010.pdf]

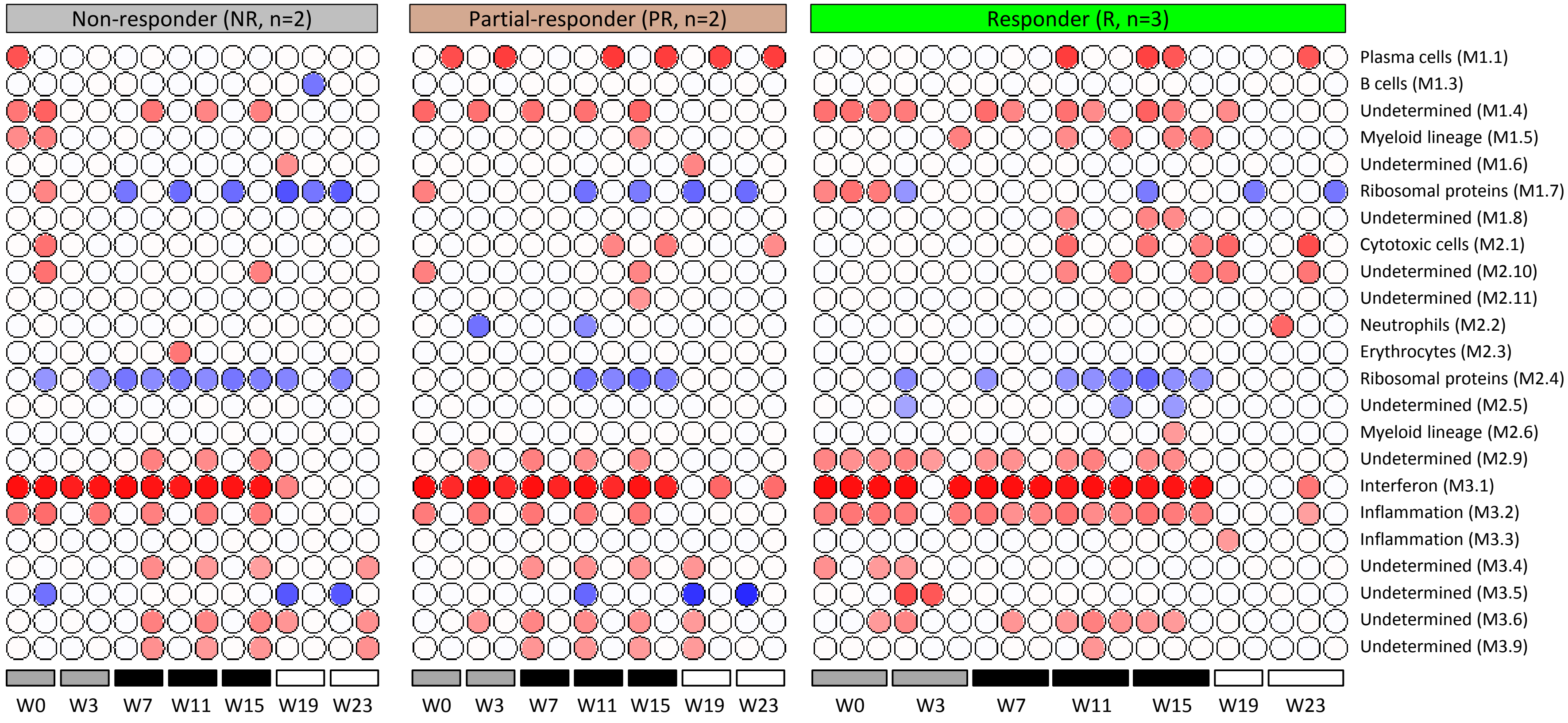
Non-responder (NR, n=2)
Partial-responder (PR, n=2)
Responder (R, n=3)
Plasma cells (M1.1)
B cells (M1.3)
Undetermined (M1.4)
Myeloid lineage (M1.5)
Undetermined (M1.6)
Ribosomal proteins (M1.7)
Undetermined (M1.8)
Cytotoxic cells (M2.1)
Undetermined (M2.10)
Undetermined (M2.11)
Neutrophils (M2.2)
Erythrocytes (M2.3)
Ribosomal proteins (M2.4)
Undetermined (M2.5)
Myeloid lineage (M2.6)
Undetermined (M2.9)
Interferon (M3.1)
Inflammation (M3.2)
Inflammation (M3.3)
Undetermined (M3.4)
Undetermined (M3.5)
Undetermined (M3.6)
Undetermined (M3.9)
W0 W3 W7 W11 W15 W19 W23
W0 W3 W7 W11 W15 W19 W23
W0 W3 W7 W11 W15 W19 W23
