# Supplementary material for: Intrahepatic Transcriptional Signature Associated with Response to Interferon-α Treatment in the Woodchuck Model of Chronic Hepatitis B
Source: PLoS Pathog. 2015 Sep 9;11(9):e1005103. doi: 10.1371/journal.ppat.1005103 (PMC4564242; doi:10.1371/journal.ppat.1005103)
Supplement: S1 Table — The liver histology score was derived from the lobular sinusoidal hepatitis score combined with the mean of the portal hepatitis score (n = 1–5 portal tracts examined). A composite histology score of >0–2 indicates mild hepatitis, >2–4 indicates moderate hepatitis and >4 indicates marked to severe hepatitis. Dashed line indicates no sample at week -3 (baseline). The LLOD for the anti-WHs assay was 100 StdU/mL. Values between 100–200 StdU/mL were considered trace; 200–300 very low; 300–500 low; 500–2,000 moderate; and greater than 2,000 were considered high (i.e. indicates potential seroconversion). Animals M1004 and F1022 had anti-WHs values >500 StdU/mL at weeks 5–25 and 13–23, respectively. The response group classifications are described in Table 1. (DOCX) [file ppat.1005103.s012.docx]

| Treatment group | Woodchuck ID# | Response group | Liver histology score: baseline/max | Max anti-WHs (StdU/mL) |
| --- | --- | --- | --- | --- |
| wIFN-α | M1002 | R | –/5.0 | 100 |
|  | M1003 | PR | 1.0/3.7 | 100 |
|  | M1004 | N/A | 2.0/3.0 | 19,128 |
|  | M1006 | N/A | 0.0/0.4 | 100 |
|  | M1007 | N/A | 1.0/4.0 | 151 |
|  | M1012 | NR | 0.0/4.7 | 369 |
|  | F1013 | R | 0.0/3.0 | 100 |
|  | F1014 | NR | –/2.0 | 164 |
|  | F1018 | PR | –/1.0 | 275 |
|  | F1020 | N/A | 0.5/5.0 | 164 |
|  | F1022 | R | 1.0/1.0 | 8,711 |
|  | F1023 | N/A | 0.0/0.5 | 100 |
| Placebo | M1001 | N/A | 0.7/1.0 | 120 |
|  | M1005 | N/A | –/3.0 | 230 |
|  | M1008 | N/A | 0.5/3.4 | 124 |
|  | M1009 | N/A | –/3.0 | 147 |
|  | M1010 | N/A | 1/2.3 | 188 |
|  | M1011 | N/A | 1.0/2.0 | 100 |
|  | F1015 | N/A | 0.4/4.4 | 100 |
|  | F1016 | N/A | 0.0/0.2 | 101 |
|  | F1017 | N/A | 0.4/1.2 | 100 |
|  | F1019 | N/A | 0.5/1.0 | 100 |
|  | F1021 | N/A | 0.4/2.0 | 100 |
|  | F1024 | N/A | 1.0/1.3 | 120 |

**S1 Table. Histologic and antibody response to wIFN-α and placebo treatment.**
