# Supplementary material for: Intrahepatic Transcriptional Signature Associated with Response to Interferon-α Treatment in the Woodchuck Model of Chronic Hepatitis B
Source: PLoS Pathog. 2015 Sep 9;11(9):e1005103. doi: 10.1371/journal.ppat.1005103 (PMC4564242; doi:10.1371/journal.ppat.1005103)
Supplement: S2 Table — Contigs were mapped with BLAST and E-value cut-off of 1.e-10 to human, mouse and rat coding and non-coding RefSeq transcripts. The numbers in parentheses in the first column are the total number of genes or transcripts in the corresponding RefSeq databases as of November 2014. The version 1 transcriptome assembly is described in reference [24]. The version 2 transcriptome assembly is described in the Methods. (DOCX) [file ppat.1005103.s013.docx]

**S2 Table. Comparison of the original (version 1) and revised (version 2) woodchuck transcriptome assemblies.**

| **Assembly Characteristics** | **Version 1** | **Version 2** |
| --- | --- | --- |
| Number of human genes (26,140) | 16,750 (64.1%) | 18,246 (69.8%) |
| Number of mouse genes (24,180) | 14,870 (61.5%) | 16,017 (66.2%) |
| Number of rat genes (17,215) | 12,221 (71.0%) | 12,954 (75.2%) |
| Unique human genes  (only in one assembly) | 694 | 2,190 |
| Unique mouse genes  (only in one assembly) | 690 | 1,837 |
| Unique rat genes  (only in one assembly) | 556 | 1,289 |
| Coverage of human transcriptome (48,627) | 35,212 (72.4%) | 37,809 (77.8%) |
| Coverage of mouse transcriptome (33,236) | 22,098 (66.5%) | 23,674 (71.2%) |
| Coverage of rat transcriptome (18,073) | 12,893 (71.3%) | 13,679 (75.7%) |
| Number of contigs  (including singletons) | 61,034 | 219,479 |
| Average contig length | 947 | 1,571 |
