# Supplementary material for: Intrahepatic Transcriptional Signature Associated with Response to Interferon-α Treatment in the Woodchuck Model of Chronic Hepatitis B
Source: PLoS Pathog. 2015 Sep 9;11(9):e1005103. doi: 10.1371/journal.ppat.1005103 (PMC4564242; doi:10.1371/journal.ppat.1005103)
Supplement: S3 Table — A total of 233 ISGs were compiled from these gene sets, of which 209 were present in version 2 of the woodchuck transcriptome. The intrahepatic expression of these ISGs in woodchucks treated with wIFN-α is displayed in Fig 7A. (DOCX) [file ppat.1005103.s014.docx]

**S3 Table. Sources of annotated ISGs for characterization of the intrahepatic IFN response in woodchucks treated with wIFN-α.**

| **ISG Set Description** | **Reference** |
| --- | --- |
| IFN module (M3.1) | Chaussabel D, Quinn C, Shen J, Patel P, Glaser C, Baldwin N, et al. A modular analysis framework for blood genomics studies: application to systemic lupus erythematosus. Immunity 2008;29:150-164 |
| Type I ISG GSEA reactome  Type II ISG GSEA reactome  IFN signaling GSEA reactome | Subramanian A, Tamayo P, Mootha VK, Mukherjee S, Ebert BL, Gillette MA, et al. Gene set enrichment analysis: a knowledge-based approach for interpreting genome-wide expression profiles. Proc Natl Acad Sci U S A 2005;102:15545-15550.  http://www.broadinstitute.org/gsea/index.jsp |
| Type I ISGs in human hepatocytes  Type II ISGs in human hepatocytes | He XS, Nanda S, Ji X, Calderon-Rodriguez GM, Greenberg HB, Liang TJ. Differential transcriptional responses to interferon-alpha and interferon-gamma in primary human hepatocytes. J Interferon Cytokine Res 2010;30:311-320. |
| IFN signaling pathway  PKR-IFN signaling pathway | Ingenuity Pathway Analysis canonical pathways  http://www.ingenuity.com/ |
