# Supplementary material for: Intrahepatic Transcriptional Signature Associated with Response to Interferon-α Treatment in the Woodchuck Model of Chronic Hepatitis B
Source: PLoS Pathog. 2015 Sep 9;11(9):e1005103. doi: 10.1371/journal.ppat.1005103 (PMC4564242; doi:10.1371/journal.ppat.1005103)
Supplement: S4 Table — qRT-PCR data expressed as fold-change relative to week -3 (pre-treatment baseline). Week 0: sample collected 6 hours post-first dose of 20 μg wIFN-α or placebo. Week 7: sample collected 6 hours post-first dose of 100 μg wIFN-α or 23rd dose of placebo. ND: not determined (animal died prior to biopsy time-point (see Table 1), insufficient sample available or mRNA quality not appropriate for analysis). Intrahepatic expression of these genes at week 0 and week 7 was not significantly different (Fig 7B). The response group classifications are described in Table 1. (DOCX) [file ppat.1005103.s015.docx]

| Treatment group | Woodchuck ID# | Response group | Mx1 Expression | | ISG15 Expression | | IRF9 Expression | | OAS1 Expression | |
| --- | --- | --- | --- | --- | --- | --- | --- | --- | --- | --- |
|  |  |  | Week 0 | Week 7 | Week 0 | Week 7 | Week 0 | Week 7 | Week 0 | Week 7 |
| wIFN-5α | M1002 | R | 27.9 | 43.1 | 13.7 | 5.4 | 6.7 | 2.9 | 7.6 | 11.9 |
|  | M1003 | PR | ND | ND | ND | ND | ND | ND | ND | ND |
|  | M1004 | N/A | ND | ND | ND | ND | ND | ND | ND | ND |
|  | M1006 | N/A | ND | ND | ND | ND | ND | ND | ND | ND |
|  | M1007 | N/A | 21.6 | 38.5 | 13.7 | 8.3 | 3.8 | 14.3 | 6.8 | 13.9 |
|  | M1012 | NR | 43.1 | 30.7 | 7.3 | 10.6 | 12.6 | 7.3 | 0.8 | 1.3 |
|  | F1013 | R | 54.6 | 97.5 | 20.8 | 44.6 | 24.0 | 41.2 | 7.5 | 22.7 |
|  | F1014 | NR | ND | 266.0 | ND | 108.2 | ND | 56.1 | ND | 34.7 |
|  | F1018 | PR | ND | ND | ND | ND | ND | ND | ND | ND |
|  | F1020 | N/A | 29.6 | 35.1 | 4.3 | 3.4 | 5.1 | 5.2 | 2.7 | 4.1 |
|  | F1022 | R | 159.8 | 172.7 | 14.1 | 14.2 | 16.7 | 18.3 | 18.6 | 51.8 |
|  | F1023 | N/A | 62.5 | ND | 4.5 | ND | 16.9 | ND | 0.8 | ND |
| Placebo | M1001 | N/A | 6.3 | 16.0 | 2.1 | 5.1 | 3.8 | 4.8 | 2.8 | 3.5 |
|  | M1005 | N/A | 0.7 | 3.7 | 0.7 | 2.8 | 0.2 | 0.5 | 0.6 | 0.7 |
|  | M1008 | N/A | 4.2 | 0.7 | 8.7 | 2.2 | 3.2 | 1.3 | 6.0 | 0.8 |
|  | M1009 | N/A | 0.8 | 0.4 | 0.6 | 0.4 | 1.3 | 0.9 | 0.9 | 0.6 |
|  | M1010 | N/A | 1.3 | ND | 1.9 | ND | 1.7 | ND | 1.0 | ND |
|  | M1011 | N/A | 0.9 | 1.5 | 0.1 | 0.3 | 0.4 | 0.5 | 0.8 | 0.5 |
|  | F1015 | N/A | 0.4 | 0.6 | 1.1 | 0.4 | 2.6 | 0.4 | 0.6 | 0.8 |
|  | F1016 | N/A | 0.3 | 1.8 | 0.2 | 1.7 | 0.6 | 2.9 | 0.1 | 1.1 |
|  | F1017 | N/A | 1.2 | 1.6 | 0.5 | 0.9 | 0.7 | 2.1 | 0.6 | 0.7 |
|  | F1019 | N/A | 5.0 | 4.0 | 2.5 | 3.2 | 4.2 | 4.7 | 2.0 | 1.5 |
|  | F1021 | N/A | 1.7 | 18.9 | 2.4 | 7.6 | 1.7 | 2.8 | 4.2 | 9.4 |
|  | F1024 | N/A | 3.6 | 3.2 | 1.0 | 0.6 | 1.5 | 1.5 | 3.8 | 2.0 |

**S4 Table. qRT-PCR quantitation of select intrahepatic ISGs.**
