# Supplementary material for: Intrahepatic Transcriptional Signature Associated with Response to Interferon-α Treatment in the Woodchuck Model of Chronic Hepatitis B
Source: PLoS Pathog. 2015 Sep 9;11(9):e1005103. doi: 10.1371/journal.ppat.1005103 (PMC4564242; doi:10.1371/journal.ppat.1005103)
Supplement: S5 Table — Gene sets (modules) were identified by unsupervised WGCNA analysis and are sorted by inverse correlation with WHsAg, i.e. module 1 has highest negative r value. Cell shaded grey if correlation (negative or positive) was statistically significant (p<0.05). WGCNA modules with >1000 genes were not included since the size precluded accurate determination of key gene signatures. (DOCX) [file ppat.1005103.s016.docx]

| **WGCNA**  **module** | **Number of genes** | **Correlation value (r)** | |
| --- | --- | --- | --- |
|  |  | **WHsAg** | **WHV DNA** |
| 1 | 187 | -0.49 | -0.55 |
| 2 | 379 | -0.40 | -0.45 |
| 3 | 499 | -0.39 | -0.40 |
| 4 | 177 | -0.39 | -0.49 |
| 5 | 229 | -0.20 | -0.19 |
| 6 | 513 | -0.13 | -0.16 |
| 7 | 58 | -0.04 | 0.02 |
| 8 | 779 | -0.02 | 0.02 |
| 9 | 141 | -0.01 | -0.01 |
| 10 | 315 | 0.08 | 0.09 |
| 11 | 352 | 0.17 | 0.17 |
| 12 | 747 | 0.23 | 0.25 |
| 13 | 128 | 0.32 | 0.38 |
| 14 | 262 | 0.43 | 0.54 |

**S5 Table. Correlation of different gene sets with WHsAg and WHV DNA.**
