# Supplementary material for: Intrahepatic Transcriptional Signature Associated with Response to Interferon-α Treatment in the Woodchuck Model of Chronic Hepatitis B
Source: PLoS Pathog. 2015 Sep 9;11(9):e1005103. doi: 10.1371/journal.ppat.1005103 (PMC4564242; doi:10.1371/journal.ppat.1005103)
Supplement: S6 Table — qRT-PCR data expressed as fold-change relative to week -3 (pre-treatment baseline). Week 0: sample collected 6 hours post-first dose of 20 μg wIFN-α or placebo. Week 7: sample collected 6 hours post-first dose of 100 μg wIFN-α or 23rd dose of placebo. Week 15: sample collected 6 hours post-last dose of 100 μg wIFN-α or 45th dose of placebo. ND: not determined (animal died prior to biopsy time-point (see Table 1), insufficient sample available or mRNA quality not appropriate for analysis). The response group classifications are described in Table 1. (DOCX) [file ppat.1005103.s017.docx]

**S6 Table. qRT-PCR quantitation of select intrahepatic genes.**

| Treatment group | Woodchuck ID# | Response group | T-bet (TBX21) Expression | | | TRAIL (TNFSF10) Expression | | | CXCL9 Expression | | |
| --- | --- | --- | --- | --- | --- | --- | --- | --- | --- | --- | --- |
|  |  |  | Week 0 | Week 7 | Week 15 | Week 0 | Week 7 | Week 15 | Week 0 | Week 7 | Week 15 |
| wIFN-α | M1002 | R | 1.5 | 1.3 | 180.6 | 9.5 | 5.7 | 17.7 | 4.7 | 11.2 | 17.0 |
|  | M1003 | PR | ND | ND | ND | ND | ND | ND | ND | ND | ND |
|  | M1004 | N/A | ND | ND | ND | ND | ND | ND | ND | ND | ND |
|  | M1006 | N/A | ND | ND | ND | ND | ND | ND | ND | ND | ND |
|  | M1007 | N/A | 0.9 | 2.4 | ND | 3.0 | 5.4 | ND | 4.7 | 3.3 | ND |
|  | M1012 | NR | 0.2 | 0.0 | 0.0 | 2.0 | 2.0 | 0.4 | 7.1 | 2.3 | 1.7 |
|  | F1013 | R | 4.4 | 6.1 | 16.0 | 2.0 | 6.2 | 4.6 | 0.1 | 88.9 | 30.9 |
|  | F1014 | NR | ND | 0.0 | 0.2 | ND | 47.6 | 118.0 | ND | 1.1 | 2.0 |
|  | F1018 | PR | ND | ND | ND | ND | ND | ND | ND | ND | ND |
|  | F1020 | N/A | 1.0 | 3.9 | 0.9 | 6.0 | 0.3 | 3.2 | 21.1 | 0.0 | 11.9 |
|  | F1022 | R | 4.5 | 0.7 | 16.9 | 0.9 | 1.0 | 89.8 | 0.5 | 1.3 | 142.8 |
|  | F1023 | N/A | 1.5 | ND | ND | 10.4 | ND | ND | 14.4 | ND | ND |
| Placebo | M1001 | N/A | 1.1 | 0.7 | 0.5 | 5.3 | 4.1 | 0.6 | 9.9 | 3.7 | 1.0 |
|  | M1005 | N/A | 4.1 | 0.7 | 1.5 | 0.4 | 2.0 | 0.3 | 0.3 | 4.0 | 22.7 |
|  | M1008 | N/A | ND | ND | ND | ND | ND | ND | ND | ND | ND |
|  | M1009 | N/A | 4.3 | 2.5 | ND | 1.2 | 0.3 | ND | 1.0 | 0.7 | ND |
|  | M1010 | N/A | 0.2 | ND | 0.2 | 0.1 | ND | 0.3 | 0.1 | ND | 1.1 |
|  | M1011 | N/A | 20.0 | 7.6 | 9.4 | 9.1 | 6.0 | 5.0 | 4.3 | 12.8 | 14.1 |
|  | F1015 | N/A | 15.7 | 2.6 | 5.9 | 0.6 | 1.0 | 0.4 | 7.2 | 29.3 | 8.1 |
|  | F1016 | N/A | 0.2 | 1.3 | 0.3 | 0.6 | 12.8 | 9.5 | 1.7 | 15.3 | 4.2 |
|  | F1017 | N/A | 0.6 | 1.2 | 0.0 | 0.5 | 1.6 | 0.5 | 0.7 | 2.0 | 0.3 |
|  | F1019 | N/A | 0.4 | 0.3 | 0.5 | 8.2 | 0.8 | 4.3 | 0.1 | 0.0 | 0.0 |
|  | F1021 | N/A | 0.3 | 0.3 | 0.1 | 8.2 | 66.2 | 7.6 | 0.9 | 11.2 | 0.0 |
|  | F1024 | N/A | 1.6 | 0.3 | ND | 2.2 | 1.3 | ND | 1.5 | 0.9 | ND |
