# Supplementary material for: Intrahepatic Transcriptional Signature Associated with Response to Interferon-α Treatment in the Woodchuck Model of Chronic Hepatitis B
Source: PLoS Pathog. 2015 Sep 9;11(9):e1005103. doi: 10.1371/journal.ppat.1005103 (PMC4564242; doi:10.1371/journal.ppat.1005103)
Supplement: S7 Table — F: forward primer; R: reverse primer; P: probe. Note the Hugo gene symbol for TRAIL is TNFSF10. (DOCX) [file ppat.1005103.s018.docx]

| Gene | Primers and Probe | Sequence |
| --- | --- | --- |
| IRF9 | F | 5’-CCCAGACTGACCGTGTATAAAG-3’ |
|  | R | 5’-AGCCACTCTCCAAACAGAAC-3’ |
|  | P | 5’-AACAATGGCTGTAGTTCCTCAGAGGG-3’ |
| ISG15 | F | 5’-AAGCACCAACAGCCTCTAC-3’ |
|  | R | 5’-GTTCTCGGCACTCTTCTGTT-3’ |
|  | P | 5’-ATGGAGCTGGAACAGCCAGCTTA-3’ |
| TRAIL | F | 5’-GCTGATGAAAAGTGCCAGAAAT-3’ |
|  | R | 5’-TTCGGTCATTTTCCTTAAGTTCA-3’ |
|  | P | 5’-TTGTTGGTCCAAAGATTCTGAA-3’ |

**S7 Table.** **Oligonucleotides used for qRT-PCR.**
